# Self-Defensive Antimicrobial Shape Memory Polyurethanes with Honey-Based Compounds

**DOI:** 10.1021/acsami.3c12274

**Published:** 2023-12-04

**Authors:** Maryam Ramezani, Emily Elizabeth Labour, Jingjing Ji, Anand Utpal Vakil, Changling Du, Thalma Kabeyi Orado, Shikha Nangia, Mary Beth Browning Monroe

**Affiliations:** Department of Biomedical and Chemical Engineering, Syracuse Biomaterials Institute, and BioInspired Syracuse: Institute for Material and Living Systems, Syracuse University, Syracuse, New York 13244, United States

**Keywords:** antimicrobial, shape memory polymer, polyurethane, phenolic acids, biofilms

## Abstract

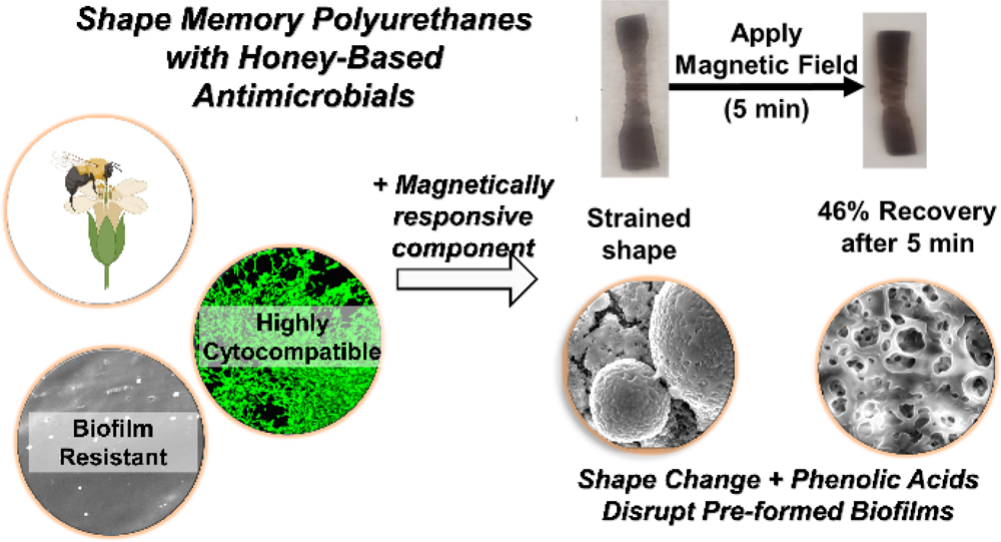

Infection treatment plays a crucial role in aiding the body in wound healing. To that end, we developed a library of antimicrobial polymers based on segmented shape memory polyurethanes with nondrug-based antimicrobials (i.e., honey-based phenolic acids (PAs)) using both chemical and physical incorporation approaches. The antimicrobial shape memory polymers (SMPs) have high transition temperatures (>55 °C) to enable maintenance of temporary, programmed shapes in physiological conditions unless a specific external stimulus is present. Polymers showed tunable mechanical and shape memory properties by changing the ratio, chemistry, and incorporation method of PAs. Cytocompatible (∼100% cell viability) synthesized polymers inhibited growth rates of *Staphylococcus aureus* (∼100% with physically incorporated PAs and >80% with chemically incorporated PAs) and *Escherichia coli* (∼100% for samples with cinnamic acid (physical and chemical)). Crystal violet assays showed that all formulations inhibit biofilm formation in surrounding solutions, and chemically incorporated samples showed surface antibiofilm properties with *S. aureus*. Molecular dynamics simulations confirm that PAs have higher levels of interactions with *S. aureus* cell membranes than *E. coli*. Long-term antimicrobial properties were measured after storage of the sample in aqueous conditions; the polymers retained their antimicrobial properties against *E. coli* after up to 20 days. As a proof of concept, magnetic particles were incorporated into the polymer to trigger user-defined shape recovery by applying an external magnetic field. Shape recovery disrupted preformed *S. aureus* biofilms on polymer surfaces. This antimicrobial biomaterial platform could enable user- or environmentally controlled shape change and/or antimicrobial release to enhance infection treatment efforts.

## 1 Introduction

The wound healing process can be influenced by infection, which is defined as an imbalance between the bacteria percentage and host tissue.^1,2^ Generally, wounds can heal if the body overcomes the bacteria invasion using a satisfactory immune response.^3^ Otherwise, the wound healing mechanism can be seriously inhibited by infection, resulting in nonhealing, chronic wounds.^4^ Chronic wounds currently affect approximately 2% of people in the US. After 4 weeks of nonhealing, an extended inflammation phase arises, which is associated with infection formation, pain/discomfort, and amputations in severe situations.^5−9^ Current clinical options for wound treatment include wound cleaning and debridement, using bandages and wound dressings, hyperbaric oxygen therapy, and skin grafts. Some of these methods can increase infection risks,^10^ while other options, such as skin grafts and advanced wound dressings, can be complicated, limited in availability, and/or expensive.^11^

In treating wound infections, traditional oral antibiotic delivery has several disadvantages, such as kidney and liver malfunction, restricted circulation into the infected target, and overall toxicity.^12,13^ Localized drug delivery from wound dressing surfaces is a useful approach that can reduce the required drug concentration to address some toxicity concerns.^14−16^ However, an added complication of wound infection is the grand challenge of antibiotic-resistant bacteria strains, caused in part by antibiotic overuse.^17^ Antibiotic-resistant infections contribute to ∼100,000 deaths and cost around $2 billion each year in the US alone.^4,18,19^ Thus, nondrug-based approaches are required to reduce these risks. In the wound care process, wound dressings can play an important role in infection protection if they cover wounds effectively to seal them from external pathogens while releasing nondrug antimicrobial agents to treat infection.^10^ Antimicrobial treatment can lower the bacteria load in the wound and allow the healing process to occur. Many commercial wound dressings contain silver. Silver had desirable antibacterial properties, but it is cytotoxic and can hinder natural wound healing processes.^20−24^ Thus, new approaches to infection control in wound care are required.

Here, we aimed to develop a self-defensive shape memory polymer (SMP) wound dressing that releases cytocompatible antimicrobial agents and could be coupled with other stimuli-responsive moieties to respond to environmental or user-defined stimuli. SMPs are smart materials that are synthesized in their primary shape with relaxed chains. Heating above their transition temperature increases chain movement and softens the material so that they can be mechanically deformed into a secondary shape with strained chains. Cooling can fix the temporary shape that is then maintained until re-exposure to another external stimulus, such as heat, enzyme release, pH change, magnetic field, or light, depending on the incorporated target within the SMP.^16,25−27^

We previously reported the synthesis of a library of tunable, biostable segmented polyurethanes with exceptional shape memory and thermomechanical properties that can maintain their programmed shape in the presence of degradation media over 40 days and then be actuated with heat to return to their original shape. Segmented polyurethanes consist of alternating hard and soft segments that play roles in shape memory and other properties. Soft segments are flexible portions with high molecular weight that confer shape change via their chain movements. Hard segments are rigid portions of the polymer backbone that are responsible for shape fixation via hydrogen bond formation. These polyurethanes can be tuned for a wide range of applications. For instance, they can be implanted in a secondary shape. Upon implantation, a specified stimulus can trigger shape recovery to deliver a range of bioactive molecules such as antimicrobials or bioactive factors. To enable a response to specific local physiological stimulus, the SMP transition temperature should be above body temperature so that heating after implantation will not trigger shape recovery. Our previously developed polyurethanes with high transition temperatures could provide a reliable platform for wound healing applications that can be coupled with safe antimicrobial agents and specific responsive moieties, including magnetic particles, light-responsive particles, peptides, or pH- and ROS-responsive agents.^28^ We previously demonstrated magnetic and bacterial-protease response in these SMPs to enable user or environmental control over shape change.^25,28,29^

To make antimicrobial wound dressing materials, we took advantage of phenolic acids (PAs). PAs are honey-based nondrug agents with proven antimicrobial properties against a range of bacterial strains; they effectively change bacterial membrane permeability and/or metabolism to hinder nutrient influx and kill bacteria.^30−32^ We previously demonstrated that scaffolds containing PAs have higher cytocompatibility compared to silver-containing foams.^33^ Thus, the incorporation of PAs into wound dressings could prevent antibiotic overuse without impeding wound healing processes.

**Figure 1:**
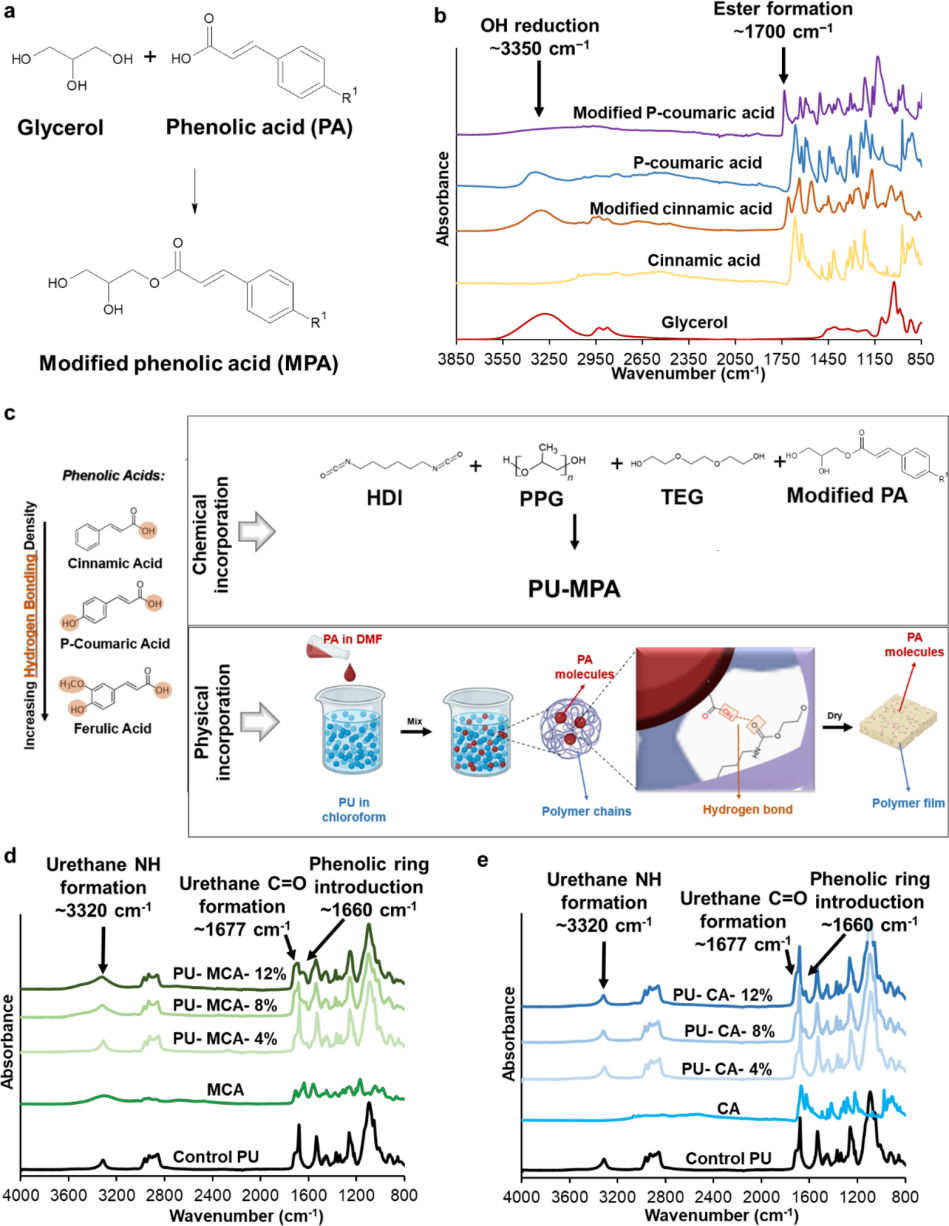
(a) Phenolic acid (PA) modification with glycerol to form modified PA (MPA). (b) Fourier transform infrared (FTIR) spectra of modified cinnamic and *p*-coumaric acids. (c) Schematic representation of processes for chemical and physical incorporation of PAs into the polyurethane (PU). FTIR spectra of (d) representative samples of PU with chemically incorporated modified cinnamic acid (MCA) and (e) representative samples of PU with physically incorporated cinnamic acid (CA).

Here, we utilized a segmented thermoplastic shape memory polyurethane with varying ratios of incorporated PAs (cinnamic acid (CA), *p*-coumaric acid (PCA), and ferulic acid (FA)). To incorporate PAs into the polyurethane system, two methods were employed: chemical and physical incorporation. For chemical incorporation, PAs were modified with glycerol via esterification (Figure 1a). In this process, one of the three OH groups in glycerol reacts with the carboxylic acid group of the PA and the other two OH groups are retained. The resulting diol can react with the other polyurethane monomers without undesired chain termination that would occur with the chemical incorporation of unmodified PAs. For physical incorporation, PAs were dissolved and physically added to synthesized polymer solutions before being cast into a film. Physical cross-links, such as hydrogen bonds, hold the incorporated PAs within polymer chains (Figure 1b).

After synthesis, we analyzed and compared shape memory, thermomechanical properties, surface chemistry, cytocompatibility, antimicrobial properties, and antibiofilm properties of synthesized polymers. We also assessed PA release over time and antimicrobial, surface, and thermal properties over extended exposure to aqueous environments. We employed molecular dynamics simulations to provide insights into how PAs interact with bacterial membranes and enable the rational design of PA-based antimicrobial approaches. In the long term, these materials could provide a nontoxic, biostable, antimicrobial wound dressing, which could reduce the overuse of antibiotics while lowering infection, pain, and healthcare costs. They could also be coupled with responsive materials to provide on-demand release of PAs upon actuation and shape recovery.^25^ As a proof of concept, we explored the effects of magnetically actuated shape change on preformed biofilm structures on SMP surfaces.

## 2 Materials and Methods

### 2.1 Materials

Polypropylene glycol (PPG, M.W. 2000 kDa), hexamethylene diisocyanate (HDI), triethylene glycol (TEG), glycerol, CA, trans PCA, FA, chloroform, dimethylformamide (DMF), phosphate-buffered saline (PBS), Dulbecco’s modified Eagle’s medium (DMEM), penicillin–streptomycin (P/S), fetal bovine serum (FBS), and crystal violet (CV) were purchased from Fisher Scientific (Waltham, Massachusetts, USA) and used as received. Ethyl-3-(3-(dimethylamino)propyl)carbodiimide (EDC), 4-(*N*,*N*-dimethylamino)pyridine (DMAP), and dibutyltin dilaurate (DBTDL) were purchased from Sigma-Aldrich (St. Louis, Missouri, USA). *Escherichia coli* (*E. coli*, 397E strain), *Staphylococcus aureus* (*S. aureus*, Wichita strain), and Swiss mouse 3T3 fibroblasts were purchased from ATCC (Manassas, Virginia, USA), and glutaraldehyde was purchased from Electron Microscopy Sciences (Hatfield, Pennsylvania, USA). PPG, TEG, EDC, DMAP, CA, PCA, FA, and glycerol were all dehydrated under a vacuum overnight to remove trace moisture before use. All solvents were dried over sieves before use.

### 2.2 Synthesis

#### 2.2.1 Esterification of Cinnamic Acid

CA (6 g) was dissolved in 60 mL of chloroform in a round-bottomed flask (RBF). Then, 0.49 g of DMAP was added and stirred for 30 min under nitrogen. Next, 3.72 g of glycerol was dissolved in 20 mL of DMF and added to the reaction on ice for 5 min, followed by addition of 7.32 g of EDC dissolved in 20 mL of chloroform. The reaction continued for 5 days at room temperature under N_2_. Every 24 h, Fourier transform infrared (FTIR) spectroscopy (Nicolet, Thermo Fisher Scientific, Waltham, Massachusetts) was ran on the solution to track ester formation. Upon completion of reaction, a rotary evaporator was utilized to remove the solvent.^34^ Synthesis of modified CA (MCA) was confirmed by using FTIR and nuclear magnetic resonance (NMR) spectroscopy (Figure S1). MCA: ^1^H NMR ((CD_3_)_2_SO; ppm): 4.4 (m, >C–C*H*_*2*_–O−), 7.4–7.5 (m, −C*H*=, aromatic).

#### 2.2.2 Esterification of *p*-Coumaric Acid

PCA (6 g) was dissolved in 60 mL of DMF in an RBF, and then 0.44 g of DMAP was added to the flask and dissolved. Glycerol (3.36 g) was dissolved in 20 mL of DMF and added to the RBF, which was placed on ice for 5 min. Then, 7.72 g of EDC dissolved in 20 mL of chloroform was added to the flask and reacted for 1 h at room temperature under N_2_. The RBF was placed in an oil bath at 50 °C and allowed to react for 5 days. Every 24 h, FTIR spectra were collected on a small amount of the mixture to track ester formation. Upon completion of the reaction, the mixture was washed with 0.1 M HCl and the organic bottom layer was collected using a separatory funnel. This layer was washed with saturated sodium bicarbonate overnight. Then, the bottom layer was collected and magnesium sulfate was added until floating crystal formation was observed. The mixture was stirred for 10 min and filtered with vacuum. The solvent was removed by using a rotary evaporator. Synthesis of modified PCA (MPCA) was confirmed using FTIR and NMR spectroscopy (Figure S1). MPCA: ^1^H NMR ((CD_3_)_2_SO; ppm): 4.4 (m, > C–C*H*_*2*_-O−), 7.4–7.5 (m, −C*H*=, aromatic).

#### 2.2.3 Control Polyurethane (PU) Synthesis

PPG (6.25 g), TEG (1.98 g), HDI (3.1 g), and DBTDL (0.1 g) were added into a speed mixer cup inside a moisture-controlled glovebox. The cup was sealed and placed in a speed mixer (FlackTek, Inc., Landrum, South Carolina) at 3500 rpm for 30 s. The mixture was then poured into a Petri dish with a Teflon liner and placed in an oven at 50 °C for 48 h. FTIR spectroscopy was carried out on the final product to verify successful synthesis.

#### 2.2.4 Chemical Incorporation of Modified Phenoxyl Acids into Control PU

PPG (6.25 g), HDI (3.01 g), and DBTDL (0.1 g) were added to a speed mixer cup with a magnetic stir bar in a moisture-controlled glovebox. The cup was sealed and placed on a hot plate at 65 °C/1200 rpm to react for 18 h. The mixture was cooled to room temperature while stirring and then transferred back to the glovebox. The required amounts of TEG and modified PA (MPA, details shown in Table 1) were dissolved in 5 mL of DMF and added to the cup. The cup was mixed at 3500 rpm for 30 s and then reacted for 1 h at 65 °C/1200 rpm. Then, the polymer mixture was poured into a Petri dish with a Teflon liner, covered with a lid, and placed in an oven at 50 °C for 48 h. Trace solvent was removed using a vacuum oven at 50 °C.

**Table 1:** Chemical Compositions of Synthesized Segmented Thermoplastic Polyurethanes with Phenolic Acids^a^

	**sample**	**HDI** (mol %)	**PPG** (mol %)	**TEG** (mol %)	PA %
control PU	control	50	10	40	
chemically incorporated phenolic acids (M: modified with glycerol)	PU-MCA-4%	50	10	36	4
	PU-MCA-8%	50	10	32	8
	PU-MCA-12%	50	10	28	12
	PU-MPCA-4%	50	10	36	4
	PU-MPCA-8%	50	10	32	8
	PU-MPCA-12%	50	10	28	12
physically incorporated phenolic acids	PU-CA-4%	50	10	40	4
	PU-CA-8%	50	10	40	8
	PU-CA-12%	50	10	40	12
	PU–PCA-4%	50	10	40	4
	PU–PCA-8%	50	10	40	8
	PU–PCA-12%	50	10	40	12
	PU-FA-4%	50	10	40	4
	PU-FA-8%	50	10	40	8
	PU-FA-12%	50	10	40	12

a PU: polyurethane; HDI: hexamethylene diisocyanate; PPG: poly(propylene glycol); TEG: triethylene glycol; PA: phenolic acid; CA: cinnamic acid; PCA: p-coumaric acid; FA: ferulic acid.

#### 2.2.5 Catalyst Removal

Synthesized polymers were dissolved in chloroform, precipitated with cold diethyl ether, and filtered using a vacuum funnel to remove the catalyst prior to further use.

#### 2.2.6 Physical Incorporation of Phenolic Acids into Control PU

Control polymer (2 g) was dissolved in 8 mL of chloroform. Selected PAs were dissolved in 2 mL of DMF at 4, 8, and 12 wt % and then added to the polymer solution. The mixture was cast into a Teflon mold and dried overnight at room temperature, followed by vacuum drying at 50 °C overnight to remove trace solvent. Table 1 summarizes the chemical composition of the synthesized polyurethanes with chemically and physically incorporated PAs.

### 2.3 Chemical and Thermomechanical Characterization

#### 2.3.1 Spectroscopic Analysis

Surface chemistry of the synthesized monomers and polymers was analyzed using an FTIR spectrometer (Nicolet iS5, Fisher Scientific, Waltham, Massachusetts, USA) at 4 cm^–1^ resolution using OMNIC software (Fisher Scientific, Waltham, Massachusetts, USA) to confirm functional groups after esterification and incorporation into PU.

#### 2.3.2 Thermal Analysis

TA Instruments Q500 was employed as a thermal gravimetric analyzer (TGA) to study the thermal degradation of synthesized polymers. Samples (5–10 mg) were placed in platinum pans and heated to 450 °C at 10 °C/min. The temperature corresponding to 3% mass loss was considered as the thermal degradation temperature and used as a guide for the maximum temperature that could be employed in further thermal processing and analysis steps. A differential scanning calorimeter (DSC) TA Q200 was then used to measure transition glass transition temperatures (*T*_g_) and melting temperatures (*T*_m_) of the polymers. Samples (3–5 mg, *n* = 3) were placed in *t*-zero aluminum pans and equilibrated at −40 °C, heated to 150 °C at 10 °C/min kept isothermally for 2 min, cooled to 50 °C at 5 °C/min, kept isothermally for 20 min, cooled to −40 °C, kept isothermally for 2 min, and finally reheated to 150 °C at 10 °C/min. The midpoint of the first endothermic inflection above 0 °C and the minimum of the melting peak in the second heating cycle were recorded as the *T*_g_ and *T*_m_ of the hard segment, respectively.^35,36^

#### 2.3.3 Mechanical Properties

Dog bones (*n* = 3; ASTM D638 scaled down by a factor of 4) were cut with a gauge length of 6.25 mm and width of 1.5 mm and placed into a tensile tester (Instron, Norwood, Massachusetts, USA) with a 24 N load cell. A strain rate of 2 mm/min was applied to the samples until failure. Young’s modulus, tensile strength, and maximum strain at fracture were determined from the resulting stress/strain curves.

#### 2.3.4 Shape Memory Behavior

TA Instruments Q800 in controlled-force mode was utilized to measure shape memory features. Dog bones (ASTM D638 type IV, scaled down by a factor of 4) with a gauge length of 6.25 mm and width of 1.5 mm were cut from prepared films. The samples were heated to 65 °C, and a controlled force was applied to stretch them to 20% strain at 0.03 N/min. In the next step, samples were cooled to −5 °C at 2 °C/min. Then, the samples were unloaded at 0.1 N/min and reheated to 65 and 2 °C/min to measure the recovery ratio. This cycle was repeated three times. Fixing ratio (*R*_f_) and recovery ratio (*R*_r_) were calculated using eqs 1 and 2, in which ϵ_u_ is the strain after unloading (the fixed temporary shape), ϵ_m_(*L*) is the maximum strain at loading, and ϵ_p_ is the remaining strain after recovering the original shape (permanent strain).
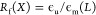
1
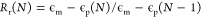
2

### 2.4 Cytocompatibility

3T3 Swiss mouse fibroblasts (ATCC-CCL92) were used to assess cytocompatibility of the samples (*n* = 3 cylinders, 6 mm diameter). Samples were washed in 20× volume deionized water and PBS while being sonicated to remove any solvent and unreacted chemicals and then sterilized using UV light for 20 min (10 min/side). 3T3 cells were cultured in a T75 flask with DMEM (containing 10% FBS and 1% PS) at 37 °C/5% CO_2_. After 3–4 days and upon reaching >85% confluency, 10,000 cells were seeded in each well of a 24-well plate and incubated for 24 h. Fresh media were added to the cells, and then sterilized samples were placed in Transwell inserts above the seeded cells.

Two-hundred microliters of 30% H_2_O_2_ served as negative control, and positive controls contained empty inserts in media with no samples. After 24 h of incubation, a Live (Calcein AM, green)/Dead (BOBO-3 Iodide, red) assay (Thermo Fisher Scientific) was utilized to evaluate sample cytocompatibility. Stains were applied to cells for 30 min at room temperature, and then cells were imaged using an inverted fluorescent microscope (three field views/sample).

A resazurin assay was also performed to measure the relative density of live cells as compared with empty insert positive controls. After 24 h of incubation with samples and media removal, 10% resazurin in media was added to the cells for 2 h. Two hundred microliters of the solution was transferred to a 96-well plate, and absorbance was measured using the plate reader (excitation: 530 nm, emission: 590 nm). Equation 4 was used for cell density quantification relative to the empty well control:
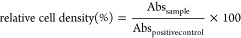
4

### 2.5 Antimicrobial Properties

#### 2.5.1 Antimicrobial Efficacy over 1 Week

To investigate the antimicrobial properties of the samples, *E. coli* and *S. aureus* were used. Samples (*n* = 3) were cut using a 6 mm biopsy punch (∼35 mg specimens, which contained ∼1.4, 2.8, and 4.2 mg PA in 4, 8, and 12% samples, respectively) from films and sterilized using a UV box for 1 h. Silver-containing foam (Areza Medical, Dallas, Texas, USA) was utilized as positive clinical control based on its previously reported antimicrobial efficacy.^30,33^

*E. coli* and *S. aureus* were cultured as previously described.^37^ Briefly, bacteria were incubated in 5 mL of autoclaved lysogeny broth (LB, 25 g/L in DI water) at 37 °C with shaking for 16 h. Then, 1 mL of the bacterial solution was transferred to 10 mL of fresh LB and incubated at 37 °C with shaking to reach the desired optical density (OD= 0.6) at 600 nm using a plate reader (FLx800, BioTek Instruments, Inc.).

Bacterial solution (200 μL) was added to each well containing sterilized samples and incubated at 37 °C/100 rpm for 1 week. Control wells included fresh LB with no bacteria and bacteria in LB without exposure to any samples. At each time point (2, 6, and 24 h and then every 24 h up to 7 days), the bacterial solution was mixed using a micropipette and 100 μL of solution from each well was transferred into a 96-well plate. Optical densities were measured by using a plate reader at 600 nm. The bacterial solution was then transferred back onto the samples to continue the experiment. Growth inhibition was calculated using eq 3. Every 2 days, 50 μL of fresh LB media was added to each well to prevent effects of evaporation. Deionized water was applied in the wells surrounding the sample wells to help maintain moisture.
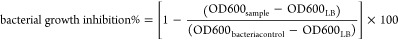
3

#### 2.5.2 Antibiofilm Ability

*S. aureus* was cultured using the method described in Section 2.5.1 until bacteria reached an OD of 0.4 at 600 nm, as previously described.^29^ Samples were sterilized using UV light for 1 h and placed in a 24-well plate with 1975 μL of autoclaved LB, 25 μL of autoclaved 20% glucose in DI water, and 400 μL of *S. aureus*. Samples were incubated at 37 °C without shaking to enable biofilm formation.^38^ The samples were removed after 24 or 48 h and rinsed with 0.1% NaCl. Bacteria were fixed by soaking samples in 2.5% glutaraldehyde for 1 h. To dry the samples, they were placed in 15, 30, 50, and 60% acetone for 15 min at each concentration and then dipped in 75 and 100% acetone and air-dried overnight at room temperature for imaging.

A Denton Vacuum sputter coater (Moorestown, New Jersey) was used to sputter-coat the samples with gold, and then a scanning electron microscope (SEM, JEOL JSM 5600, Tokyo, Japan) was used to image biofilm structures on samples at 1500× magnification and a voltage of 20 kV.

To quantify the biomass within the surrounding the well plate, a CV assay was employed based on the method described by Mahmoudi et al.^39,40^ Briefly, bacterial solution was removed from each well and biofilms were washed three times using DI water. Then, 200 μL of 0.1% CV in DI water was added to each well and incubated for 20 min at room temperature. The stain was removed carefully without disrupting the biofilm, and excess CV was washed away using 2 mL of DI water three times. The wells were dried overnight at 37 °C, and 2 mL of 30% acetic acid was added to dissolve the CV at 37 °C/100 rpm for 30 min. Then, 200 μL of each solution was transferred into a 96-well plate and absorbance was measured using a plate reader at 560 nm. Wells containing only LB, bacteria, and glucose were used as negative controls, and Ag foams were used as positive controls.

### 2.6 Computational Methods

#### 2.6.1 Atomistic to Coarse-Grain Mapping

The atomistic structures of the three PAs (CA, PCA, and FA) were obtained from Automated Topology Builder (ATB) and Repository.^41,42^ A single molecule of each PA was solvated in SPCE water,^43^ followed by energy minimization, 20 ns of isothermal–isochoric (*NVT*) equilibration at 310 K, 20 ns of isothermal–isobaric (*NPT*) equilibration steps, and 50 ns production molecular dynamics (MD) runs using the GROMACS software suite.^44^ The PyCGTOOL was used to generate the coarse-grained (CG) model parameters of the equilibrated structures of the three PAs.^45^ CA was mapped using SC5 and P1 beads; PCA was mapped using SC4, SP1, and P1 beads; and FA was mapped using SC4, SP1, Na, and P1 beads, based on Martini_v2.2 force field definitions (Figure S2 and Table S1).^46−49^ Lipid A in the outer leaflet and 1,2-dipalmitoyl-*sn*-glycero-3-phosphoethanolamine (DPPE) in the inner leaflet were used to mimic the *E. coli* outer membrane. The peptidoglycan scaffold was used to mimic the *S. aureus* membrane. The initial CG membranes of *E. coli* and peptidoglycan surfaces of *S. aureus* were built using a locally modified version of Python script called insane.py,^50^ which has been reported in our earlier work.^51,52^

#### 2.6.2 CG Molecular Dynamics (CGMD) Simulations

The initial simulation system was created by placing a 9 × 9 nm^2^ patch of *E. coli* membrane in the *xy*-plane in the center of a 9 × 9 × 20 nm^3^ simulation box. In the + *z* direction above the membrane plane, 40 CA molecules were placed randomly, avoiding spatial overlap with the membrane and among the CA molecules. The insane.py script was used to add MARTINI water and Na^+^ to neutralize the system.^50^ Additional Na^+^ and Cl^–^ were added to maintain a 0.085 M ionic concentration of the solution. A similar process was repeated for *S. aureus* peptidoglycan system in which the box size was 12 × 12 × 26 nm^3^.

The MARTINI (version 2.2) CG force field was used in this work.^46−49^ All CG MD simulations were performed using GROMACS version 2019.^44^ The systems were simulated using a four-step process: (i) energy minimization using the steepest-descent algorithm; (ii) isothermal–isochoric (NVT) equilibration (200 ns) using the velocity-rescale thermostat with a coupling constant τ_t_ = 1.0 ps and *T* = 310 K;^53^ (iii) semi-isotropic *NPT* equilibration (200 ns) with pressure coupling at 1 bar using a Berendsen barostat with a coupling constant τ_p_ = 12.0 ps and compressibility of 4.5 × 10^–5^ bar^–1^ and *T* = 310 K;^54^ and (iv) a 2 μs production run with pressure coupling at 1 bar using a Parrinello–Rahman barostat with τ_p_ = 12.0 ps and compressibility of 4.5 × 10^–5^ bar^–1^ and *T* = 310 K.^55^ A 20 fs time step was used, and the nonbonded interaction neighbor list was updated every 20 steps. A 1.1 nm cutoff was used for electrostatic and van der Waals interactions. The electrostatic screening constant (ε_r_) value of 15 was used for the MARTINI water model. Three-dimensional periodic boundary conditions were applied to each system. The interaction energies between 40 CAs and each bacterial membrane type were computed by defining energy groups for acids and membranes in the GROMACS input file. Similar setup and CGMD simulations were performed for PCA and FA with *E. coli* and *S. aureus*.

### 2.7 Property Retention over Time

#### 2.7.1 Antimicrobial Property Retention

To characterize the retention of antimicrobial properties, samples (*n* = 3, ∼35 mg) were incubated in 5 mL of sterile PBS at 37 °C for up to 30 days. At 5, 10, 20, and 30 days of PBS incubation, a subset of samples were removed from the media and antimicrobial properties were tested using the method described in Section 2.5.1 at 2, 6, 24, 48, and 72 h.

#### 2.7.2 Mass Loss

A second set of samples was used in a mass loss study in PBS at 37 °C. At days 5 and 10, samples were removed, washed with DI water, and dried under vacuum. Then, a scale was used to measure mass relative to initial mass before storage in PBS. Sacrificial samples were used for surface and thermal analyses using FTIR and DSC, respectively, at the same time points as described above.

#### 2.7.3 Surface Hydrophobicity

A goniometer (Model 500, Ramé-hart Co, Succasunna, New Jersey) was used to measure the contact angle of water on samples at 0, 5, and 10 days of storage in PBS at 37 °C. To that end, 0.2 mL water droplets were placed onto the films and 100 images were captured at 0.01 s intervals using a SuperSpeed U4 series camera. DROPimage software was used for image analysis to determine the angle between the material surface and the water droplet. An average of 100 measurements was used to determine the contact angle of the samples with three replicates.

### 2.8 Magnetically Responsive Antimicrobial Polyurethanes

As a proof of concept, Fe_3_O_4_ nanoparticles synthesized as previously described^28^ were incorporated into a PU-CA-8% film by mixing in at 24 wt % prior to casting in a Teflon mold. After trace chloroform was removed using a vacuum oven, small rectangles (15 × 4 mm) were punched from the film. To prepare strained samples, we heated them to 75 °C and manually stretched them before cooling them to room temperature to fix their temporary shape. The initial and final lengths were measured by using digital calipers. Strained samples without magnetic nanoparticles incorporated were used as a shape-changing control, and nonstrained samples with and without magnetic nanoparticles were used as static controls. After sterilizing samples, the biofilm assay described in Section 2.5.2 was performed to grow biofilm on sample surfaces for 24 h. The samples were washed and exposed to an alternating magnetic field (strength: 0.5 mT; frequency: 5 Hz) for 5 min. Sample dimensions were measured after magnetic field exposure using digital calipers, and samples were imaged by using a camera. Samples were washed, and remaining bacteria were fixed on the surface using method described in Section 2.5.2. SEM was used to visualize the biofilm on the polymer surfaces.

### 2.9 Statistical Analysis

Data are presented as the mean ± standard deviation. Sample sizes are provided, with data presented in each figure. ANOVA with *post hoct* test was performed using Microsoft Excel. Statistical significance was taken as *p* < 0.05.

## 3 Results and Discussion

### 3.1 Spectroscopic Analysis

Figure 1a shows the reaction scheme employed to modify PAs, and Figure 1b displays the FTIR spectra of MPAs after esterification. Ester formation was confirmed by the peak at ∼1700 cm^–1^ corresponding with the C=O of the ester, and OH reduction was confirmed at ∼3350 cm^–1^ to indicate successful modification of PAs with glycerol. FTIR was also employed to characterize PA incorporation into the polymer, as shown in Figure 1c. After chemical incorporation of CA into the PU backbone, shoulders appear around 1550–1650 cm^–1^, which are associated with incorporated CA (Figure 1d).^56^ Additionally, chemical incorporation of PAs reduced the C=O of the urethane peak relative to that of the control PU. As the PA ratio increases, this reduction becomes more apparent. Spectra in Figure 1e demonstrate physical incorporation of CA into the control PU with shoulders at ∼1650 cm^–1^ to confirm incorporation. Similar results were observed in FTIR spectra of other chemically and physically incorporated samples (Figure S3).^20^

### 3.2 Thermomechanical Properties

Tables 2 and 3 show hard segment *T*_g_, *T*_m_, and Δ*H* measured using DSC (Figure S4); tensile strength, Young’s modulus, and elongation at break determined from tensile testing (Figure S5); and shape memory properties assessed using DMA (Figure S6). We chose to modify CA and PCA with glycerol via esterification (vs directly incorporating PAs using their carboxylic acid groups)) in efforts to improve mechanical properties by providing more functional groups to aid in propagation of the polymer chain. In this approach, we react free OHs of glycerol on MPAs with PU monomers to prevent unwanted chain termination by reactions between the COOH groups on CA and PCA and isocyanates. Direct incorporation of CA and PCA resulted in highly brittle polymers that did not exhibit shape memory properties (Table S2).

**Table 2:** Thermomechanical Properties of Synthesized PUs with Chemically Incorporated PAs^a^

	**thermal properties**			**mechanical properties**			**shape memory properties**
**sample**	*T*_**g**_**(°C)**	*T*_**m**_**(°C)**	**Δ***H*(J/g)	**tensile strength (kPa)**	elongation at break (%)	**modulus (kPa)**	***R**_**f**_***and *R***_**r**_**(%)**
control PU	57 ± 1	87 ± 1	12 ± 1	7600 ± 300	950 ± 2	98 ± 7	84 and 95
PU-MCA-4%	57 ± 1	81 ± 1*	7 ± 1*	5000 ± 230*	200 ± 10*	109 ± 3*	83 and 93
PU-MCA-8%	60 ± 1	87 ± 1	0.26 ± 0.1*	2200 ± 180*	187 ± 14*	33 ± 7*	88 and 95
PU-MCA-12%	60 ± 1*	89 ± 2	0.5 ± 0.2*	1500 ± 200*	208 ± 9*	23 ± 5*	91 and 100
PU-MPCA-4%	57 ± 1	89 ± 1*	16 ± 1*	7200 ± 500	800 ± 40*	187 ± 7*	64 and 97
PU-MPCA-8%	57 ± 3			5700 ± 1000*	1100 ± 140	27 ± 5*	70 and 93
PU-MPCA-12%	56 ± 4			4500 ± 600*	900 ± 70*	24 ± 1*	68 and 97

a Thermal properties are reported for the hard segment endothermic transitions observed by differential scanning calorimetry. Mean ± standard deviation displayed. *N* = 3 for mechanical, and thermal properties, *N* = 1 for shape fixity (*R*_f_) and shape recovery (*R*_r_) measurements. MCA: modified cinnamic acid; MPCA: modified *p*-coumaric acid. *p < 0.05 relative to control PU.

**Table 3:** Thermomechanical Properties of Synthesized PUs with Physically Incorporated PAs^a^

	**thermal properties**			**mechanical properties**			**shape memory properties**
**sample**	*T*_**g**_**(°C)**	*T*_**m**_**(°C)**	**Δ***H*(J/g)	**tensile strength (kPa)**	elongation at break (%)	**modulus (kPa)**	***R***_**f**_**and *R***_**r**_**(%)**
control PU	57 ± 1	87 ± 1	12 ± 1	7600 ± 300	950 ± 2	98 ± 7	84 and 95
PU-CA-4%	57 ± 1	86 ± 1*	9.2 ± 0.1*	7700 ± 500	1000 ± 60	67 ± 2*	81 and 93
PU-CA-8%	57 ± 1	86 ± 1	8.4 ± 0.2*	4600 ± 500*	700 ± 130	48 ± 7*	85 and 95
PU-CA-12%	57 ± 1	84 ± 1*	7.7 ± 0.1*	4300 ± 70*	540 ± 60*	46 ± 1*	83 and 100
PU–PCA-4%	77 ± 1*	88 ± 1*	0.5 ± 0.1*	4500 ± 460*	350 ± 40*	56 ± 5*	81 and 95
PU–PCA-8%	68 ± 1*	86 ± 1	6 ± 1*	4800 ± 300*	620 ± 70*	62 ± 3*	86 and 99
PU–PCA-12%	63 ± 7*			1500 ± 100*	59 ± 2*	56 ± 2*	0 and 0
PU-FA-4%	56 ± 1	88 ± 1*	9 ± 1*	2600 ± 60*	120 ± 20*	72 ± 1*	75 and 92
PU-FA-8%	56 ± 1	86 ± 1*	5 ± 1*	1400 ± 160*	77 ± 14*	44 ± 1*	84 and 95
PU-FA-12%	82 ± 1*			1100 ± 130*	59 ± 9*	42 ± 4*	78 and 94

a Thermal properties are reported for the hard segment endothermic transitions observed via differential scanning calorimetry. Mean ± standard deviation displayed. *N* = 3 for mechanical, and thermal properties, *N* = 1 for shape fixity (*R*_f_) and shape recovery (*R*_r_) measurements. CA: cinnamic acid; PCA: *p*-coumaric acid; FA: ferulic acid. *p < 0.05 relative to control PU.

All PUs with chemically incorporated PAs have high hard segment *T*_g_’s (above 37 °C) that are comparable to that of the control PU hard segment *T*_g_ of 57 °C (Table 2). A high *T*_g_ theoretically enables these polymers to maintain their temporary shape after implantation, unless a specific stimulus is presented to trigger shape recovery. Hard segment *T*_m_’s were similar to those of the control, but Δ*H* values decreased with increased MCA content. Similarly, the 4% MPCA sample had a comparable hard segment *T*_m_ to that of the control PU and increased Δ*H*, but 8 and 12% MPCA exhibited a loss in crystallinity with no measurable *T*_m_ or Δ*H*. We hypothesize that at higher concentrations of the bulky MPCA pendant groups, chains are unable to efficiently pack into crystals, resulting in amorphous polymers.

Increasing the MCA and MPCA content from 4 to 12% reduced the tensile strength and modulus of the resulting polymers, which correlates with reduced chain packing and crystallinity with the addition of bulky pendent groups. In the PU-MPCA polymers, tensile strength, elongation at break, and modulus were generally higher than those of corollary PU-MCA polymers, which was expected due to the extra OH in the PCA structure that serves as a hydrogen bonding site between polymer chains. The sample with 4% MPCA had mechanical properties comparable to those of the control. We hypothesize that at this concentration of MPCA, the additional hydrogen bonding sites from PCA balance out the increased chain bulkiness and reduced crystallinity to reduce the effects of PA incorporation on mechanical properties.

The shape memory properties were generally maintained compared with the control PU, with MCA samples having comparable shape fixity and recovery. There were some reductions in fixity in MPCA samples that correlate with a loss in crystallinity in these samples, but the shape recovery was comparable to that of the control.

Table 3 shows the thermomechanical properties of PUs with physically incorporated PAs. In terms of thermal properties, CA incorporation did not change hard segment *T*_g_ or *T*_m_, and it resulted in a slight but consistent decrease in Δ*H*. Incorporation of PCA increased hard segment *T*_g_, which we hypothesize is due to PCA acting as a physical cross-linker between chains with two hydrogen bonding sites. Lower concentrations of PCA did not affect hard segment *T*_m_ but did decrease Δ*H*, while 12% PCA samples were amorphous. In the crystalline regions of the polymer, unmodified PCA can physically separate chains to reduce crystallinity. Lower concentrations of FA did not affect hard segment *T*_g_ or *T*_m_ value but did reduce Δ*H*. High concentrations of FA (12%) increased hard segment *T*_g_, which again is attributed to FA acting as a physical cross-linker. In these samples, no crystallinity was observed, indicating that FA inhibited crystal formation.

In general, increasing concentrations of physically incorporated PAs decreased tensile strength, elongation, and modulus of the polymers, which corresponds with observed reductions in crystallinity. Physical incorporation did not largely affect shape memory properties, of PUs, except in the case of 12% PCA, in which we observed no shape memory properties, corresponding to its high brittleness.

### 3.3 Property Changes over Time of Incubation in PBS

**Figure 2:**
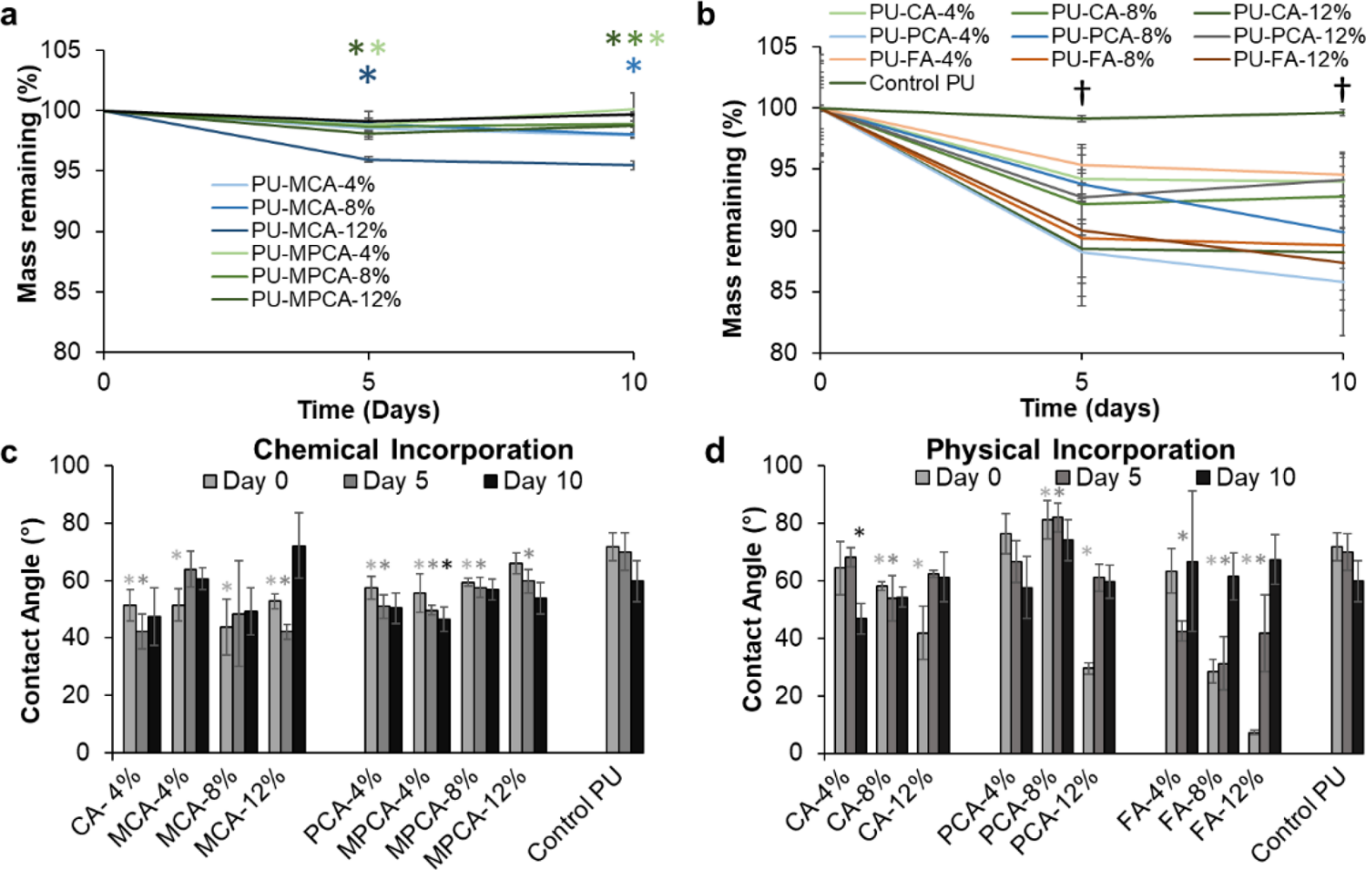
Mass remaining of polyurethanes with (a) chemically and (b) physically incorporated phenolic acids over 10 days of storage in PBS. *N* = 3, mean ± standard deviation is displayed. Contact angle changes over up to 10 days of storage in PBS for (c) chemically and (d) physically incorporated samples. *N* = 3, mean ± standard deviation displayed. *p < 0.05 relative to control PU at each time point, with font colors matching corollary sample line colors. †*p* < 0.05 relative to control PU for all phenolic acid-containing samples.

Figure 2a,b (and Figure S7) shows the mass remaining of PUs with chemically and physically incorporated PAs over 10 days. The control PU exhibited essentially 0% mass loss due to its high biostability, which was expected based on our previous work.^28^ Thus, measured mass losses were considered to be primarily due to PA release during storage in PBS at 37 °C. As expected, physical incorporation (Figure 2b) led to higher mass loss compared to chemical incorporation (Figure 2a), since physically bound PAs can be released more easily than chemically bound PAs. Based on Figure 2b, increasing the concentration of incorporated PAs generally resulted in higher mass loss, with the exception of PU–PCA-4%, which had the highest measured mass loss. This result may be due to reduced physical interaction opportunities at lower PCA content to enable its faster release.

As shown in Figure 2c, chemical incorporation of PAs decreased the initial contact angle. This result is attributed to the addition of hydrophilic ester bonds between glycerol and the PAs and/or the reduced crystallinity after PA incorporation, which increase access of water to polymer chains.^57^ Physical incorporation of PAs had larger effects on contact angle than chemical incorporation (Figure 2d), and the changes over time of storage in PBS are more significant. In general, increasing concentrations of physically incorporated PAs reduced hydrophobicity, which correlates with reduced crystallinity. These reductions were larger for FA, which might be due to more hydrogen bonding sites in its structure, enabling more interactions with water droplets. In samples with initially high hydrophilicity, the contact angle increased over time to levels that were closer to that of the control PU as PAs were released.

**Figure 3:**
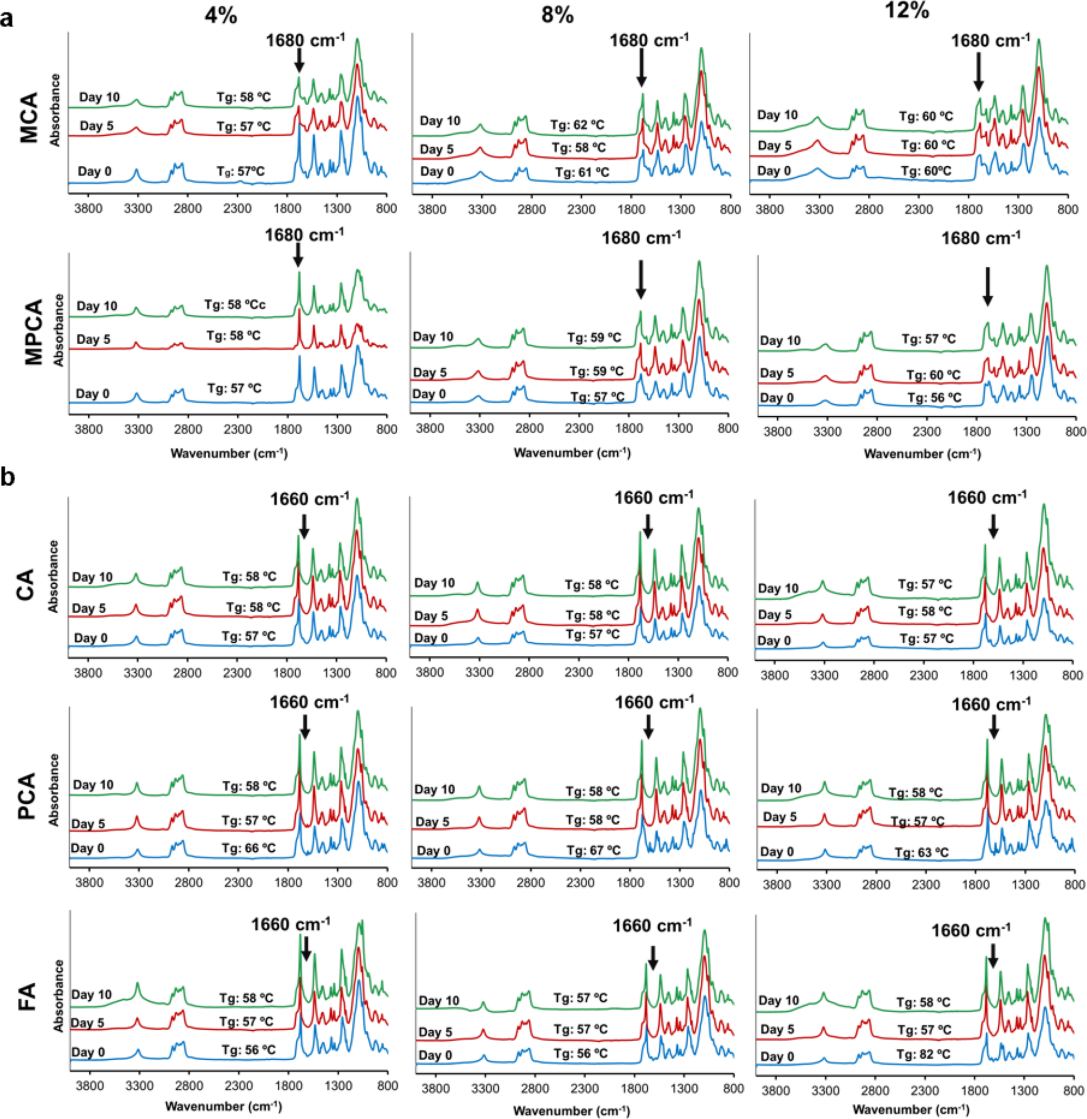
FTIR spectra and corresponding hard segment *T*_g_ (mean, *N* = 3) of (a) chemically and (b) physically incorporated samples throughout 10 days of storage in PBS.

Figure 3a and Figure S7 show the FTIR spectra and thermal properties of chemically incorporated samples at days 0, 5, and 10 of incubation in PBS. As shown, hard segment *T*_g_ and surface chemistry do not vary in these samples over this time period, corresponding to the minimal observed mass loss. For physically incorporated PUs, Figure 3b, PCA (all concentrations) and 12% FA formulations had initially higher hard segment *T*_g_’s. In these samples, PA release decreased hard segment *T*_g_ to values that are comparable to that of the control PU (∼58 °C). FTIR spectra of the physically incorporated samples over time are consistent with the mass loss and DSC results, as we can track a decrease in peaks corresponding to the PAs (1680 and 1660 cm^–1^ for chemical and physical incorporation, respectively) from days 0 to 10, indicating release of PAs into PBS.^20^

### 3.4 Cytocompatibility

**Figure 4:**
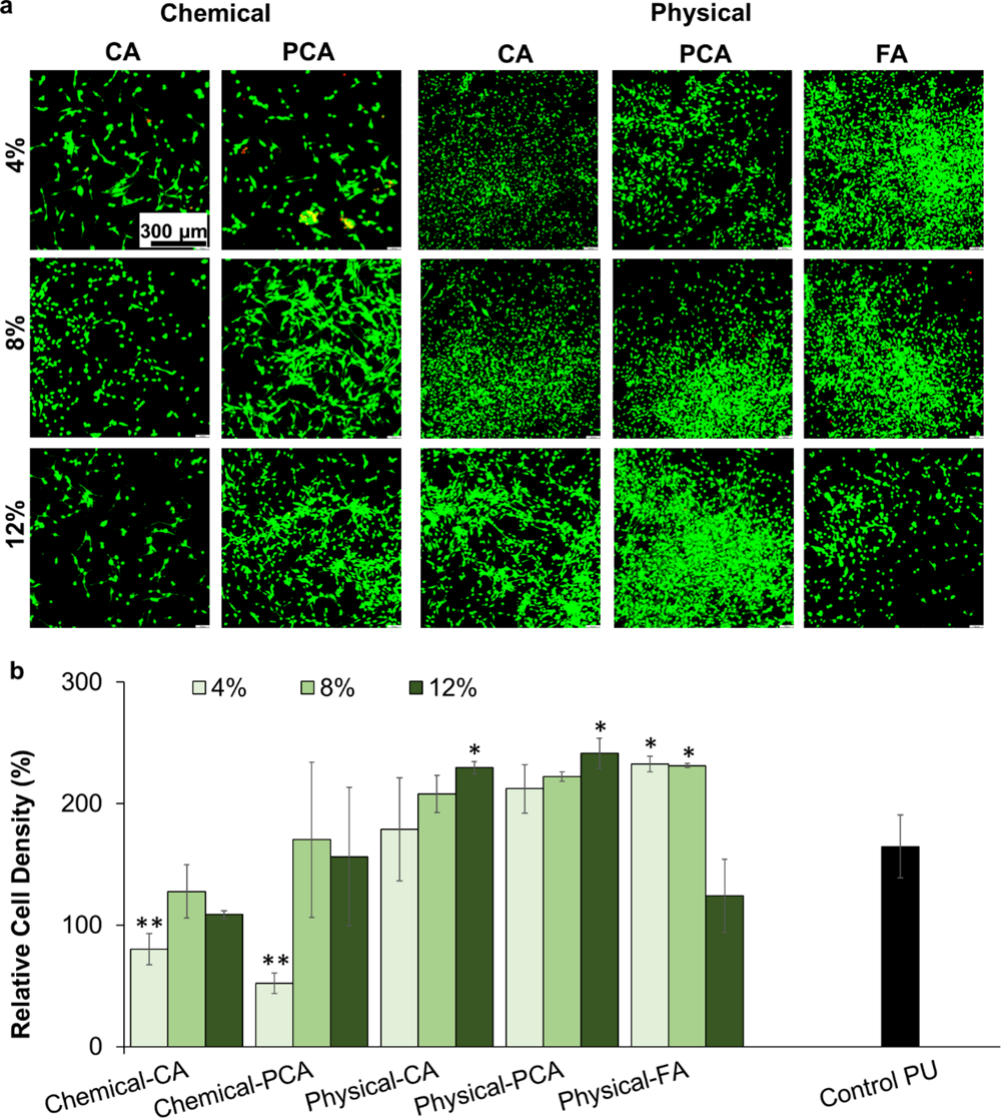
Cytocompatibility of synthesized polymers measured using (a) live/dead (scale bar applies to all images) and (b) resazurin assays after 24 h of incubation with 3T3 fibroblasts. Resazurin assay cell density is relative to cell numbers in an empty well. *N* = 3, mean ± standard deviation displayed. *p < 0.05 and **p < 0.01 relative to the control PU.

Figure 4 shows images of live and dead 3T3 cells and resazurin assay quantification of relative cell density after 24 h of incubation with samples. Most of the synthesized samples have very high cytocompatibility compared to the positive control. As PA concentration increases (specifically for physical incorporation), the cell viability increases. This result is in agreement with Ru et al., who previously reported an improvement in cell viability upon addition of PAs into their system.^58^ Harish Nayaka et al. also reported that PAs exhibit cytoprotection for 3T3 cells due to their antioxidant properties that can effectively prevent oxidative cell death.^59^ On the other hand, as shown in Figure 4b, physical incorporation of high concentrations (12%) of FA led to a significant drop in relative cell density compared to the other samples. This result correlates with a higher observed FA release (Figure 2), which may affect the pH of the cell medium to reduce cytocompatibility. Our previous work showed that Ag-based clinical control foams have poor cytocompatibility (∼30%),^33^ which is supported by work from others showing that silver can hinder healing.^20^ Thus, these highly cytocompatible PUs with incorporated PAs could be excellent substitutes for silver-based dressings with the potential to improve natural wound healing process while preventing infections.

### 3.5 Antimicrobial Properties

**Figure 5:**
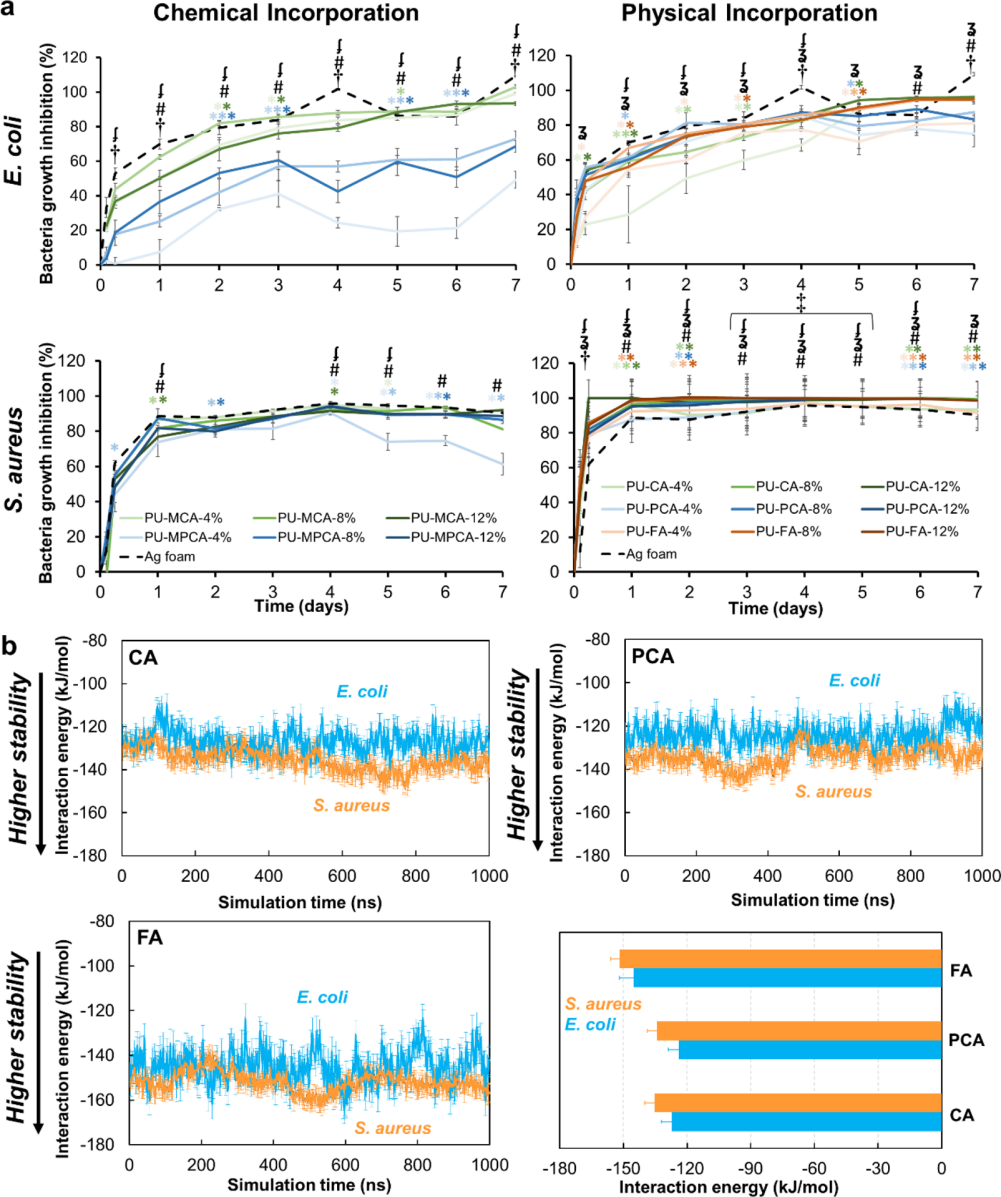
(a) Bacterial growth inhibition rate over 1 week for *E. coli* (top) and *S. aureus* (bottom) after exposure to polyurethanes with chemically (left) and physically (right) incorporated phenolic acids. *N* = 3, mean ± standard deviation displayed. Chart legends apply to all charts in a given column. *p < 0.05 relative to Ag foam at a given time point, with font colors matching corollary sample line colors, ^†^*p* < 0.05 for all phenolic acid-containing samples relative to Ag foam at a given time point. ^‡^*p* < 0.05 for all 8 and 12% phenolic acid-containing samples to relative to Ag foam at a given time point. ^‡^*p* < 0.05 between all CA concentrations at a given time point, **#***p* < 0.05 between all PCA concentrations at a given time point, and (curly three)*p* < 0.05 between all FA concentrations at a given time point. (b) Interaction energies between *E. coli* (cyan) and *S. aureus* (orange) and CA, PCA, and FA in the last 1000 ns simulations, with average interaction energies between bacteria and each phenolic acid (lower right).

Figure 5a shows the antimicrobial properties of samples (growth inhibition rate) over 7 days against *E. coli* (top row) and *S. aureus* (bottom row). Ag-based foam dressings were used as a positive clinical control due to their promising antimicrobial properties in our previous work.^30,33^ Accordingly, Ag foam showed strong antimicrobial properties against *E. coli* (∼70%) and *S. aureus* (∼90%) after 24 h of incubation, which was maintained over the 7-day study.

In the case of chemically incorporated MCA, all samples showed excellent results after 7 days, with comparable inhibition of both bacterial strains to that of the Ag foam positive control. *E. coli* growth inhibition gradually increased over time, while *S. aureus* exhibited an early and consistently high growth inhibition over the full 7 days. Chemical incorporation of MPCA had reduced effects against *E. coli* as compared to MCA, with an apparent MPCA concentration dependence. Chemically incorporated MPCA had similar growth inhibition rates to those of MCA against *S. aureus*, with a slight reduction after 5 days in the PU-MPCA-4% samples. The improved results with MCA may be due to a lack of functional hydroxyl groups on the ring; in MPCA, the pendant hydroxyl may react with monomers during synthesis to reduce PCA activity.^37^

Physically incorporated samples show promising results over 7 days as well (90–98% growth inhibition of *E. coli* and *S. aureus*) with fewer differences observed based on PA structure or concentration and more consistently high growth inhibition across all samples. Comparing *E. coli* and *S. aureus* curves over 7 days, it can be concluded that PA incorporation is more effective against *S. aureus* than *E. coli*. This trend was also observed in our previous work, which focused on characterization of PAs in solution.^37^ Using a library of 10 PAs, we measured the concentration required to kill 50% of the bacteria (IC_50_). The IC_50_ values of CA, PCA, and FA for *E. coli* were all ∼3.5 mg/mL, while those for *S. aureus* were all ∼4.5 mg/mL. Thus, lower concentrations of PAs are required to kill *E. coli* vs *S. aureus*. However, when we analyzed the log reductions in the two bacterial populations after exposure to set concentrations (5 mg/mL) of the PAs, they were on the whole higher for *S. aureus*, showing that PAs are more effective at eradicating *S. aureus* than *E. coli* when exposure concentrations are above the IC_50_ values. Additionally, we explored the effects of modifying the carboxylic acid groups on a selection of PAs, including CA and PCA. The modification did not impact efficacy against bacteria as compared with unmodified control PAs, but these experiments again showed larger log reductions of *S. aureus* as compared with *E. coli*.

To better understand these trends, we employed the computational modeling of PA interactions with the two bacterial membranes. Figure 5b shows how the interaction energies between bacteria (*E. coli* or *S. aureus*) and each type of PA evolve during the last 1000 ns of the simulations. In the MARTINI force field used in this work, the effect of nonbonded interactions, including hydrogen bonds between molecules, is accounted for. Based on this computational analysis, the interactions are stronger with *S. aureus* than with *E. coli* for all three PAs, demonstrating that PAs (aromatic pendant group) may in fact be more effective against *S. aureus*. We found that CA and PCA had comparable average interaction energies against both *E. coli* (−127.1 vs −123.9 kJ/mol for CA and PCA, respectively) and *S. aureus* (−135.4 vs −134.2 kJ/mol for CA and PCA, respectively). In comparison, FA had more negative interaction energies, corresponding to higher interaction stability (−145.1 kJ/mol with *E. coli* and −151.8 kJ/mol with *S. aureus*). This result indicates that the CH_3_–O– group on FA may contribute to increased antibacterial properties compared with CA and PCA.

Interestingly, we did not observe this effect experimentally with the PU–PAs. We attribute this outcome to the reduced FA release due to higher numbers of interaction (i.e., hydrogen bonding) sites between the PU backbone and FA molecules (Figure 2b). Lower levels of released FA correlate with reduced effects on the surrounding bacteria in the growth inhibition studies. Thus, when selecting PAs for incorporation into biomaterials to impart antimicrobial properties, interactions of PA with both the polymer backbone and the bacterial membrane must be considered.

### 3.6 Antimicrobial Property Retention

**Figure 6:**
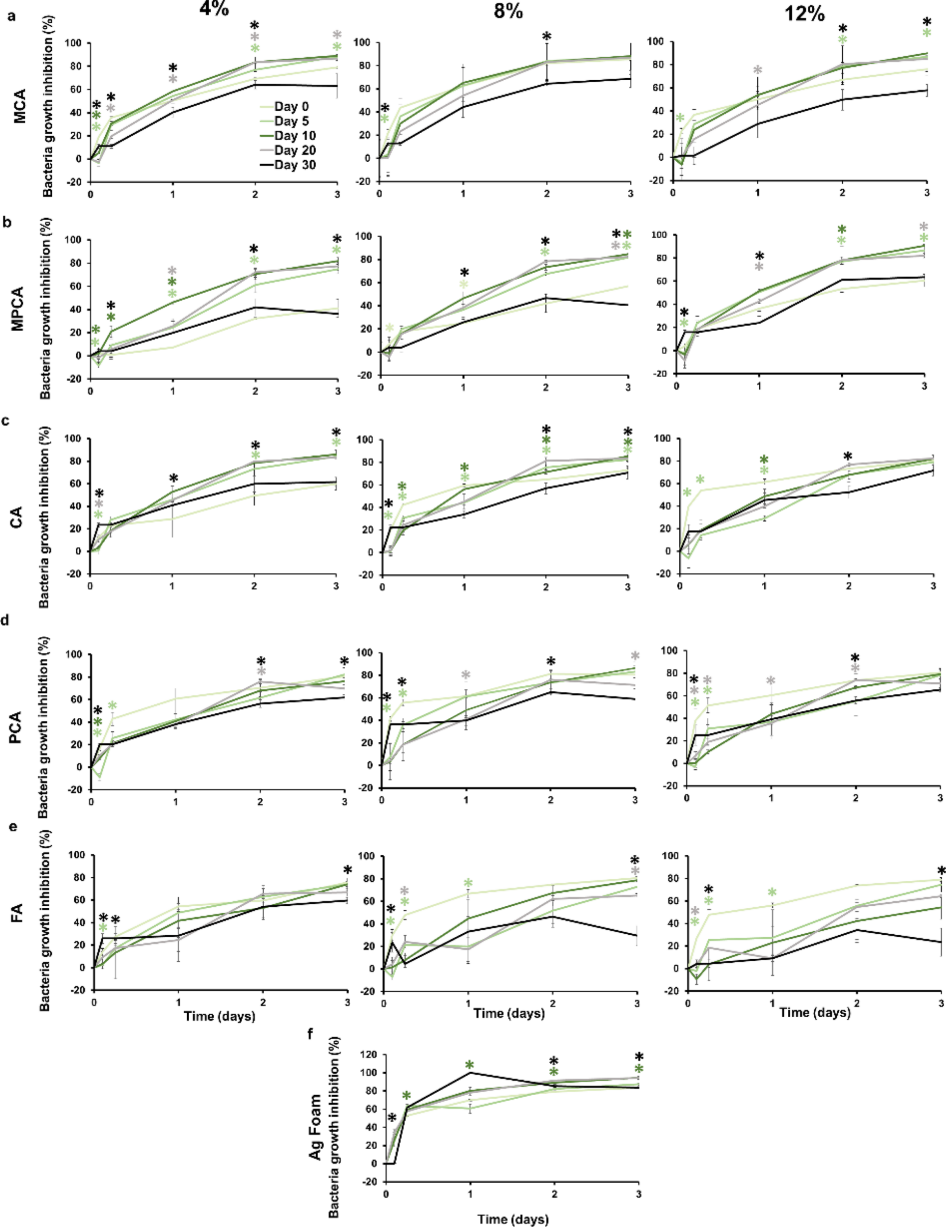
Bacterial growth inhibition by polyurethane samples with 4% (left), 8% (middle), and 12% (right) phenolic acid incorporation after 5, 10, 20, and 30 days of storage in PBS for chemically incorporated (a) CA and (b) PCA; physically incorporated (c) CA, (d) PCA, and (e) FA; and (f) Ag foam clinical controls. *N* = 3, mean ± standard deviation displayed. Chart legend applies to all charts. *p < 0.05 for given storage day relative to that at previous storage time point with font colors matching corollary sample line colors.

Figure 6 shows the *E. coli* growth inhibition rates over 3 days for samples after 5, 10, 20, and 30 days of storage in PBS to evaluate potential longer-term effects of PA release over time. Growth inhibition rates after 3 days of incubation with *E. coli* are between 60 and 80% for most samples after up to 20 days of storage. At the 30-day storage time point, inhibition at 3 days drops to below 60%, with larger decreases observed in most of the physically incorporated samples. Similar trends were reported in hydrogel materials in a previous work.^30^ This drop is most significant for physically incorporated FA in the PU-FA-12% sample. We hypothesize that this change could be due to the higher hydrophilicity and water solubility of FA, which results in higher release rates from the polymer at later time points. Ag foams maintained their antimicrobial properties for up to 30 days of incubation in PBS and show ∼80% growth inhibition at day 3 after all storage time frames (Figure 6f).

### 3.7 Antibiofilm Ability

**Figure 7:**
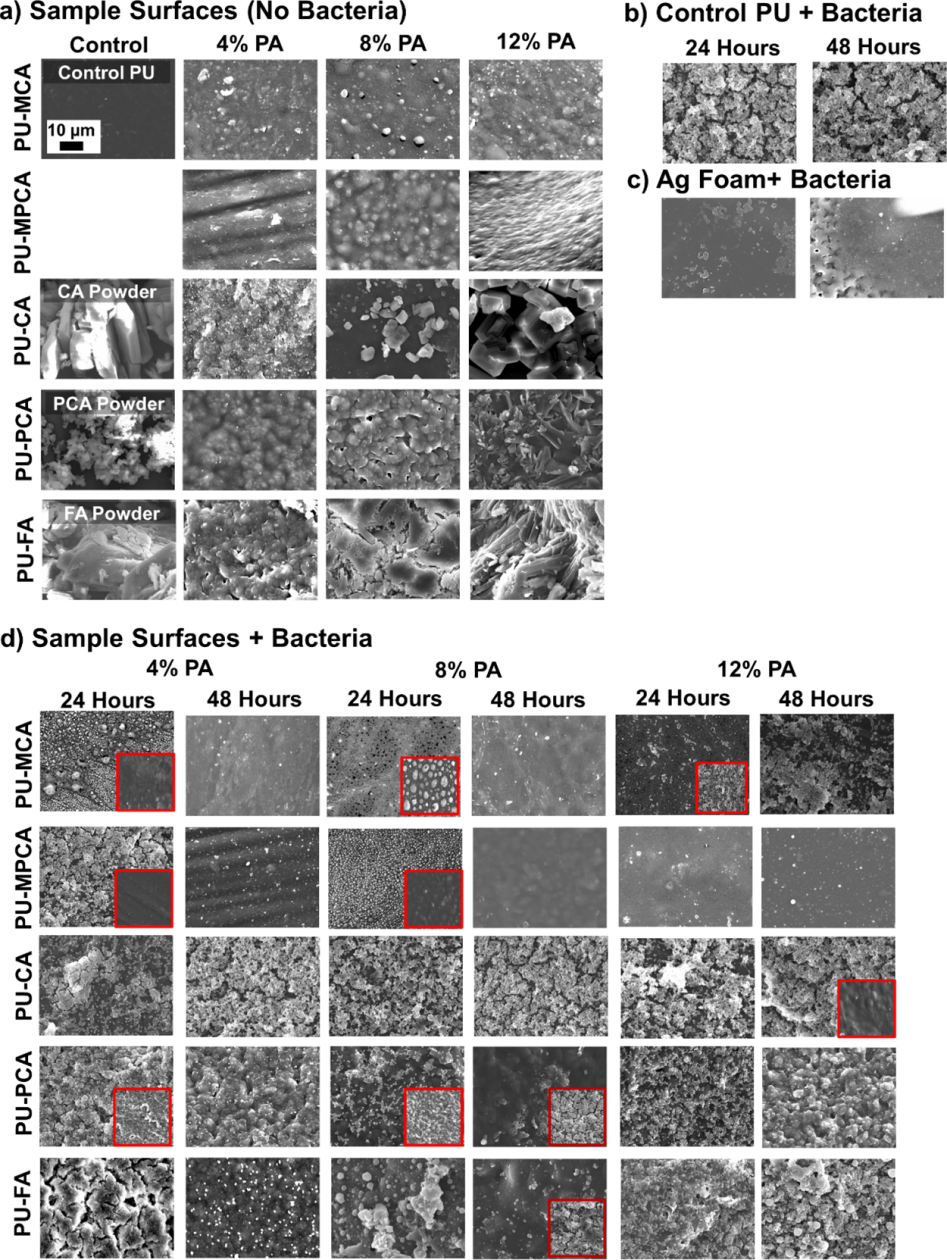
SEM images of (a) phenolic acid powder and sample surfaces before incubation with bacteria. *S. aureus* biofilm formation on surface of (b) control polyurethane (PU), (c) control Ag foam, and (d) phenolic acid-containing PU surfaces after 24 and 48 h of incubation. Red squares are small spots with varied biofilm coverage compared with the majority of the imaged surface.

SEM images of sample surfaces as synthesized (i.e., without bacteria), along with images of PA powder samples, are provided in Figure 7a to enable comparisons of effects of bacterial attachment on surfaces. As PA content increases in polymer films, surface roughness qualitatively increases, particularly in physically incorporated samples with higher (i.e., 12%) PA concentrations, which show similar crystal-like morphology to the PA powders. Control PU samples after exposure to bacteria (Figure 7b) have a thick layer of biofilms after 24 and 48 h, as shown by us previously,^29^ while only planktonic bacteria are visible on Ag foam surfaces (Figure 7c).

After chemical incorporation of modified PAs, 4% MCA and 4 and 8% MPCA samples had visible biofilm coverage by 24 h; however, antibiofilm properties are observed at 48 h on these three sample types, indicating that biofilms formed in the first 24 h were broken down between 24 and 48 h. Additionally, biofilm inhibition was observed at both 24 and 48 h for higher concentrations (12%) of MPCA (Figure 7d). These samples effectively prevented biofilm formation, resulting in planktonic bacteria attaching to the surfaces. In contrast, 12% MCA incorporation resulted in biofilm resistance at 24 h, followed by an increase in biofilm formation between 24 and 48 h. With this single exception, increasing MPA content and incubation time generally enables biofilm inhibition in these PUs.

Figure 7d reveals that physical incorporation of PAs into this PU system is less effective at preventing biofilm formation on the surface compared with chemical incorporation. Among all physically incorporated samples, 4% FA, 8% FA, and 8% PCA reduced biofilm formation to some extent at 48 h. All other remaining samples had thick layers of bacteria within protective biofilm matrices on their surfaces at both time points. Thus, the chemically incorporated samples with higher PA content generally exhibit comparable reductions in surrounding bacterial numbers with improved biofilm inhibition capabilities on the surface. Figure S8 shows the results of the CV assay quantification of the surrounding biomass in wells with samples. All PU samples reduced surrounding biofilm formation, but no clear trends emerged between sample compositions and surrounding biofilm formation.

**Figure 8:**
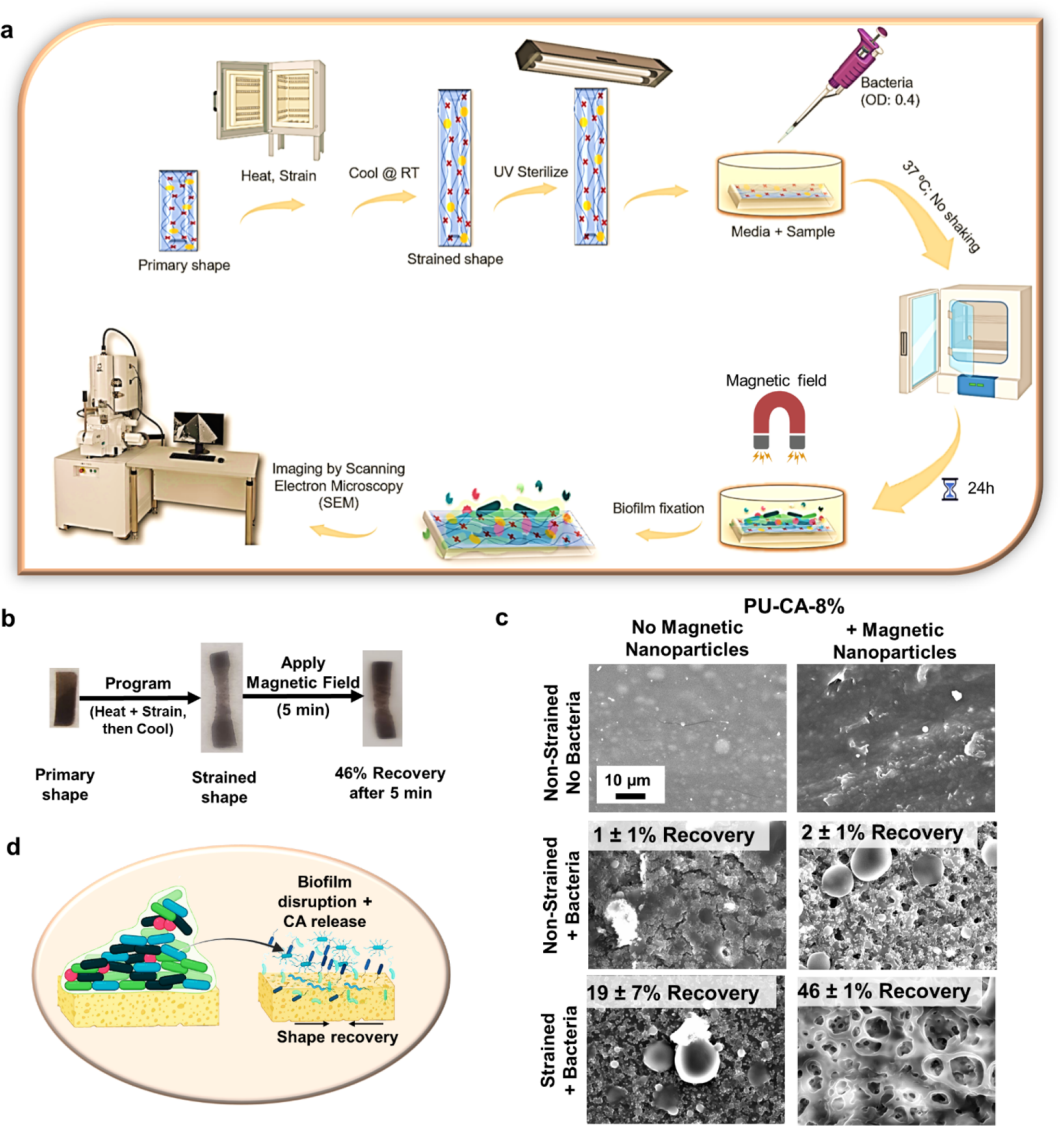
(a) Schematic representation of the biofilm assay process followed by application of magnetic field (created with BioRender.com). (b) Macroscopic view of strained nanoparticle-incorporated PU-CA-8% sample before and after biofilm assay and magnetic field application. (c) SEM images of control (no nanoparticle) and nanoparticle-incorporated PU-CA-8% samples without bacteria, nonstrained samples with bacteria, and strained samples with bacteria after *S. aureus* biofilm formation assay and 5 min magnetic field application, with measured sample shape recovery percentages overlaid. *N* = 3, mean ± standard deviation displayed. (d) Schematic representation of biofilm disruption following shape recovery and CA release (created with BioRender.com).

To explore the value of the shape memory properties with this system, we performed a biofilm assay following magnetic field actuation of magnetically responsive SMPs, as shown in Figure 8a. This study was based on previous research showing that shape change of SMPs can disrupt and/or prevent biofilm formation.^29,38,60^ In this proof-of-concept experiment, we incorporated magnetic nanoparticles into PU-CA-8%, which has robust biofilm formation (Figure 7d). Application of an alternating magnetic field causes vibration of the nanoparticles within the polymer and generates localized heating to trigger shape change in strained samples.^25,28^ Visible recovery (∼46%) of nanoparticle-containing PU-CA-8% was observed after magnetic field application, as shown in Figure 8b. While biofilm remnants remained on the nanoparticle-containing PU-CA-8% surface after triggering shape change, preliminary evidence of mechanical disruption of biofilm structures was observed, as evidenced by a lack of visible bacterial cells and the formation of large holes in the biofilms (Figure 8c). We hypothesize that the combination of a dynamic surface and potential CA release upon shape change contributed to these effects (Figure 8d). Samples with no or low recovery (unstrained and non-nanoparticle-containing, respectively) retained robust biofilms after magnetic field application, indicating that the dynamic surface contributed to the observed effects rather than the magnetic field. This property should be explored in more detail in future studies, but this experiment provides preliminary evidence that if there is another driving force for biofilm disruption, even physical incorporation of PAs—which is more straightforward—may be effective at killing isolated bacteria on the surface after shape recovery.

## 4 Conclusions

The aim of this study was to provide a biostable segmented polyurethane with antimicrobial properties from plant-based components that can be used as a substitute for common antibiotics. This material could serve as a reliable platform for incorporation of stimulus-responsive moieties to enable response to specific external stimuli such as bacteria, light, or magnetic fields. All synthesized polymers showed transition temperatures well above body temperature, which could enable secondary shape stability after implantation. Furthermore, the mechanical and shape memory properties can be tuned based on the PA incorporation method, chemistry, and concentration.

In general, physical incorporation of PAs increased growth inhibition of *E. coli* and *S. aureus* as compared with chemically incorporated samples, with higher efficacy against *S. aureus* shown experimentally and computationally. Among chemically incorporated PAs, cinnamic acid showed higher inhibition of bacterial growth compared to PCA. All samples were able to retain their antimicrobial properties for at least 10 days of storage in PBS, with larger losses in function at later time points for physically incorporated samples due to lower stability of these molecules within the films. Chemical incorporation of PAs enhanced antibiofilm properties with *S. aureus* compared to physically incorporated samples. However, if there is a driving force for biofilm disruption via biomaterial shape change, physical incorporation can be effective in destabilizing preformed biofilms. Overall, this antimicrobial SMP system could provide an effective and tunable wound dressing material that can be coupled to other stimuli-responsive components to enhance infection treatment efforts.

## Supporting Information Available

Chemical characterization of glycerol modification with phenolic acids; atomistic to CG mapping scheme; surface chemistry characterization; thermal analysis of polyurethanes; mechanical analysis of polyurethanes; shape memory properties of polyurethanes; thermomechanical, shape memory, and degradation properties of polyurethanes with direct chemical incorporation of unmodified CA and PCA; and quantification of biomass (PDF)

## References

[ref1] Pillsbury H. C. III; Kveton J. F.; Sasaki C. T.; Frazier W. Quantitative Bacteriology in Adenoid Tissue. Otolaryngol. Neck Surg. 1981, 89 (3), 355–363. 10.1177/019459988108900301. 6791091

[ref2] Zilberman M.; Elsner J. J. Antibiotic-Eluting Medical Devices for Various Applications. J. Controlled Release 2008, 130 (3), 202–215. 10.1016/j.jconrel.2008.05.020. 18687500

[ref3] Campton-Johnston S. M.; Wilson J. A. Infected Wound Management: Advanced Technologies, Moisture-Retentive Dressings, and Die-Hard Methods. Crit. Care Nurs. Q. 2001, 24 (2), 64–77. 10.1097/00002727-200108000-00009. 11858424

[ref4] Sen C. K.; Gordillo G. M.; Roy S.; Kirsner R.; Lambert L.; Hunt T. K.; Gottrup F.; Gurtner G. C.; Longaker M. T. Human Skin Wounds: A Major and Snowballing Threat to Public Health and the Economy. Wound Repair Regen. 2009, 17 (6), 763–771. 10.1111/j.1524-475X.2009.00543.x. 19903300 PMC2810192

[ref5] Werdin F.; Tenenhaus M.; Rennekampff H.-O. Chronic Wound Care. Lancet 2008, 372, 1860 10.1016/S0140-6736(08)61793-6. 19041788

[ref6] Järbrink K.; Ni G.; Sönnergren H.; Schmidtchen A.; Pang C.; Bajpai R.; Car J. The Humanistic and Economic Burden of Chronic Wounds: A Protocol for a Systematic Review. Syst. Rev. 2017, 6 (1), 15 10.1186/s13643-016-0400-8. 28118847 PMC5259833

[ref7] Chronic Wound Care Market Size, Share | Global Industry Trends [2027] https://www.fortunebusinessinsights.com/industry-reports/chronic-wound-care-market-100222.

[ref8] Pemayun T. G. D.; Naibaho R. M.; Novitasari D.; Amin N.; Minuljo T. T. Risk Factors for Lower Extremity Amputation in Patients with Diabetic Foot Ulcers: A Hospital-Based Case–Control Study. Diabet. Foot Ankle 2015, 6 (1), 29629 10.3402/dfa.v6.29629. 26651032 PMC4673055

[ref9] Bahmaninejad P.; Ghafourian S.; Mahmoudi M.; Maleki A.; Sadeghifard N.; Badakhsh B. Persister Cells as a Possible Cause of Antibiotic Therapy Failure in Helicobacter Pylori. Jgh Open 2021, 5 (4), 493–497. 10.1002/jgh3.12527. 33860100 PMC8035453

[ref10] Holmes S. P.; Rivera S.; Hooper P. B.; Slaven J. E.; Que S. K. T. Hydrocolloid Dressing versus Conventional Wound Care after Dermatologic Surgery. JAAD Int. 2022, 6, 37–42. 10.1016/j.jdin.2021.11.002. 34993497 PMC8713109

[ref11] Boulton A. J. M.; Vileikyte L.; Ragnarson-Tennvall G.; Apelqvist J. The Global Burden of Diabetic Foot Disease. Lancet 2005, 366 (9498), 1719–1724. 10.1016/S0140-6736(05)67698-2. 16291066

[ref12] Price J. S.; Tencer A. F.; Arm D. M.; Bohach G. A. Controlled Release of Antibiotics from Coated Orthopedic Implants. J. Biomed. Mater. Res. An Off. J. Soc. Biomater. Japanese Soc. Biomater. 1996, 30 (3), 281–286. 10.1002/(SICI)1097-4636(199603)30:3<281::AID-JBM2>3.0.CO;2-M. 8698690

[ref13] Ruszczak Z.; Friess W. Collagen as a Carrier for On-Site Delivery of Antibacterial Drugs. Adv. Drug Delivery Rev. 2003, 55 (12), 1679–1698. 10.1016/j.addr.2003.08.007. 14623407

[ref14] Springer B. D.; Lee G.-C.; Osmon D.; Haidukewych G. J.; Hanssen A. D.; Jacofsky D. J. Systemic Safety of High-Dose Antibiotic-Loaded Cement Spacers after Resection of an Infected Total Knee Arthroplasty. Clin. Orthop. Relat. Res. 2004, 427, 47–51. 10.1097/01.blo.0000144476.43661.10. 15552135

[ref15] Zalavras C. G.; Patzakis M. J.; Holtom P. Local Antibiotic Therapy in the Treatment of Open Fractures and Osteomyelitis. Clin. Orthop. Relat. Res. 2004, 427, 86–93. 10.1097/01.blo.0000143571.18892.8d. 15552142

[ref16] Xiao X.; Zhao W.; Liang J.; Sauer K.; Libera M. Self-Defensive Antimicrobial Biomaterial Surfaces. Colloids Surfaces B Biointerfaces 2020, 192, 110989 10.1016/j.colsurfb.2020.110989. 32361372 PMC7308212

[ref17] Zhang S.; Ge G.; Qin Y.; Li W.; Dong J.; Mei J.; Ma R.; Zhang X.; Bai J.; Zhu C.; Zhang W.; Geng D. Recent Advances in Responsive Hydrogels for Diabetic Wound Healing. Mater. Today Bio 2023, 18, 100508 10.1016/j.mtbio.2022.100508. PMC9729074 36504542

[ref18] Thorpe K. E.; Joski P.; Johnston K. J. Antibiotic-Resistant Infection Treatment Costs Have Doubled since 2002, Now Exceeding $2 Billion Annually. Health Aff. 2018, 37 (4), 662–669. 10.1377/hlthaff.2017.1153. 29561692

[ref19] Norouzi M.; Mahmoudi M.; Maleki A.; Ghafourian S. Novel Antimicrobial Target in Acinetobacter Baumannii. Clin. Lab. 2022, 68 (5), 2305–2308. 10.7754/Clin.Lab.2021.210728. 35536074

[ref20] Khansa I.; Schoenbrunner A. R.; Kraft C. T.; Janis J. E. Silver in Wound Care-Friend or Foe?: A Comprehensive Review. Plast. Reconstr. surgery. Glob. open 2019, 7 (8), e2390–e2390. 10.1097/GOX.0000000000002390. PMC6756674 31592393

[ref21] Kramer S. A. Effect of Povidone-Iodine on Wound Healing: A Review. J. Vasc. Nurs. 1999, 17 (1), 17–23. 10.1016/S1062-0303(99)90004-3. 10362983

[ref22] Goldenheim P. D. An Appraisal of Povidone-Iodine and Wound Healing. Postgrad Med. J. 1993, 69 (3), S97–105. 8290466

[ref23] Morsi N. M.; Abdelbary G. A.; Ahmed M. A. Silver Sulfadiazine Based Cubosome Hydrogels for Topical Treatment of Burns: Development and in Vitro/in Vivo Characterization. Eur. J. Pharm. Biopharm. 2014, 86 (2), 178–189. 10.1016/j.ejpb.2013.04.018. 23688805

[ref24] Lu S.; Gao W.; Gu H. Y. Construction, Application and Biosafety of Silver Nanocrystalline Chitosan Wound Dressing. Burns 2008, 34 (5), 623–628. 10.1016/j.burns.2007.08.020. 18226459

[ref25] Vakil A. U.; Ramezani M.; Monroe M. B. B. Magnetically Actuated Shape Memory Polymers for On-Demand Drug Delivery. Materials (Basel). 2022, 15 (20), 7279 10.3390/ma15207279. 36295344 PMC9611458

[ref26] Lendlein A.; Kelch S. Shape-Memory Effect from Permanent Shape. Angew. Chem., Int. Ed. Engl. 2002, 41, 2034–2057. 10.1002/1521-3773(20020617)41:12<2034::AID-ANIE2034>3.0.CO;2-M. 19746597

[ref27] Vakil A. U.; Petryk N. M.; Du C.; Howes B.; Stinfort D.; Serinelli S.; Gitto L.; Ramezani M.; Beaman H. T.; Monroe M. B. B. In Vitro and in Vivo Degradation Correlations for Polyurethane Foams with Tunable Degradation Rates. J. Biomed. Mater. Res. Part A 2023, 111 (5), 580–595. 10.1002/jbm.a.37504. 36752708

[ref28] Ramezani M.; Monroe M. B. B. biostable Segmented Thermoplastic Polyurethane Shape Memory Polymers for Smart Biomedical Applications. ACS Appl. Polym. Mater. 2022, 4 (3), 1956–1965. 10.1021/acsapm.1c01808.

[ref29] Ramezani M.; Monroe M. B. B. Bacterial Protease-Responsive Shape Memory Polymers for Infection Surveillance and Biofilm Inhibition in Chronic Wounds. J. Biomed. Mater. Res. Part A 2023, 111 (7), 921–937. 10.1002/jbm.a.37527. 36869686

[ref30] Vakil A. U.; Ramezani M.; Monroe M. B. B. Antimicrobial Shape Memory Polymer Hydrogels for Chronic Wound Dressings. ACS Appl. Bio Mater. 2022, 5 (11), 5199–5209. 10.1021/acsabm.2c00617. PMC9682482 36257053

[ref31] Dietrich H.; Nikfardjam M. S. P. Influence of Phenolic Compounds and Tannins on Wine-Related Microorganisms. In Biology of Microorganisms on Grapes, in Must and in Wine; König H., Unden G., Fröhlich J., Eds.; Springer International Publishing: Cham, 2017; pp 421–454. 10.1007/978-3-319-60021-5_18.

[ref32] Greene C.; Beaman H. T.; Stinfort D.; Ramezani M.; Monroe M. B. B. Antimicrobial PVA Hydrogels with Tunable Mechanical Properties and Antimicrobial Release Profiles. J. Funct. Biomater. 2023, 14 (4), 234 10.3390/jfb14040234. 37103324 PMC10146720

[ref33] Du C.; Liu J.; Fikhman D. A.; Dong K. S.; Monroe M. B. B. Shape Memory Polymer Foams With Phenolic Acid-Based Antioxidant and Antimicrobial Properties for Traumatic Wound Healing. Front. Bioeng. Biotechnol. 2022, 10, 809361 10.3389/FBIOE.2022.809361. 35252129 PMC8893234

[ref34] Monroe M. B. B.; Easley A. D.; Grant K.; Fletcher G. K.; Boyer C.; Maitland D. J. Multifunctional Shape-Memory Polymer Foams with Bio-Inspired Antimicrobials. ChemPhysChem 2018, 19 (16), 1999–2008. 10.1002/cphc.201701015. 29282877

[ref35] Chen T. K.; Shieh T. S.; Chui J. Y. Studies on the First DSC Endotherm of Polyurethane Hard Segment Based on 4,4‘-Diphenylmethane Diisocyanate and 1,4-Butanediol. Macromolecules 1998, 31 (4), 1312–1320. 10.1021/ma970913m.

[ref36] Choi T.; Weksler J.; Padsalgikar A.; Runt J. Microstructural Organization of Polydimethylsiloxane Soft Segment Polyurethanes Derived from a Single Macrodiol. Polymer (Guildf). 2010, 51 (19), 4375–4382. 10.1016/j.polymer.2010.07.030.

[ref37] Liu J.; Du C.; Beaman H. T.; Monroe M. B. B. Characterization of Phenolic Acid Antimicrobial and Antioxidant Structure–Property Relationships. Pharmaceutics 2020, 12 (5), 419 10.3390/pharmaceutics12050419. 32370227 PMC7285200

[ref38] Lee S. W.; Gu H.; Kilberg J. B.; Ren D. Sensitizing Bacterial Cells to Antibiotics by Shape Recovery Triggered Biofilm Dispersion. Acta Biomater. 2018, 81, 93–102. 10.1016/j.actbio.2018.09.042. 30267885 PMC6231961

[ref39] Mahmoudi M.; Sadeghifard N.; Maleki A.; Yeo C. C.; Ghafourian S. RelBE Toxin-antitoxin System as a Reliable Anti-biofilm Target in Pseudomonas Aeruginosa. J. Appl. Microbiol. 2022, 133 (2), 683–695. 10.1111/jam.15585. 35445489

[ref40] Alhusseini L. B.; Maleki A.; Kouhsari E.; Ghafourian S.; Mahmoudi M.; Al Marjani M. F. Evaluation of Type II Toxin-Antitoxin Systems, Antibiotic Resistance, and Biofilm Production in Clinical MDR Pseudomonas Aeruginosa Isolates in Iraq. Gene Reports 2019, 17, 100546 10.1016/j.genrep.2019.100546.

[ref41] Malde A. K.; Zuo L.; Breeze M.; Stroet M.; Poger D.; Nair P. C.; Oostenbrink C.; Mark A. E. An Automated Force Field Topology Builder (ATB) and Repository: Version 1.0. J. Chem. Theory Comput. 2011, 7 (12), 4026–4037. 10.1021/ct200196m. 26598349

[ref42] Koziara K. B.; Stroet M.; Malde A. K.; Mark A. E. Testing and Validation of the Automated Topology Builder (ATB) Version 2.0: Prediction of Hydration Free Enthalpies. J. Comput. Aided. Mol. Des. 2014, 28 (3), 221–233. 10.1007/s10822-014-9713-7. 24477799

[ref43] Schmid N.; Eichenberger A. P.; Choutko A.; Riniker S.; Winger M.; Mark A. E.; van Gunsteren W. F. Definition and Testing of the GROMOS Force-Field Versions 54A7 and 54B7. Eur. Biophys. J. 2011, 40 (7), 843–856. 10.1007/s00249-011-0700-9. 21533652

[ref44] Abraham M. J.; Murtola T.; Schulz R.; Páll S.; Smith J. C.; Hess B.; Lindahl E. GROMACS: High Performance Molecular Simulations through Multi-Level Parallelism from Laptops to Supercomputers. SoftwareX 2015, 1–2, 19–25. 10.1016/j.softx.2015.06.001.

[ref45] Graham J. A.; Essex J. W.; Khalid S. PyCGTOOL: Automated Generation of Coarse-Grained Molecular Dynamics Models from Atomistic Trajectories. J. Chem. Inf. Model. 2017, 57 (4), 650–656. 10.1021/acs.jcim.7b00096. 28345910

[ref46] de Jong D. H.; Singh G.; Bennett W. F. D.; Arnarez C.; Wassenaar T. A.; Schäfer L. V.; Periole X.; Tieleman D. P.; Marrink S. J. Improved Parameters for the Martini Coarse-Grained Protein Force Field. J. Chem. Theory Comput. 2013, 9 (1), 687–697. 10.1021/ct300646g. 26589065

[ref47] Periole X.; Marrink S.-J. The Martini Coarse-Grained Force Field. Methods Mol. Biol. 2013, 924, 533–565. 10.1007/978-1-62703-017-5_20. 23034762

[ref48] Marrink S. J.; Risselada H. J.; Yefimov S.; Tieleman D. P.; de Vries A. H. The MARTINI Force Field: Coarse Grained Model for Biomolecular Simulations. J. Phys. Chem. B 2007, 111 (27), 7812–7824. 10.1021/jp071097f. 17569554

[ref49] Monticelli L.; Kandasamy S. K.; Periole X.; Larson R. G.; Tieleman D. P.; Marrink S.-J. The MARTINI Coarse-Grained Force Field: Extension to Proteins. J. Chem. Theory Comput. 2008, 4 (5), 819–834. 10.1021/ct700324x. 26621095

[ref50] Wassenaar T. A.; Ingólfsson H. I.; Böckmann R. A.; Tieleman D. P.; Marrink S. J. Computational Lipidomics with Insane: A Versatile Tool for Generating Custom Membranes for Molecular Simulations. J. Chem. Theory Comput. 2015, 11 (5), 2144–2155. 10.1021/acs.jctc.5b00209. 26574417

[ref51] Ma H.; Cummins D. D.; Edelstein N. B.; Gomez J.; Khan A.; Llewellyn M. D.; Picudella T.; Willsey S. R.; Nangia S. Modeling Diversity in Structures of Bacterial Outer Membrane Lipids. J. Chem. Theory Comput. 2017, 13 (2), 811–824. 10.1021/acs.jctc.6b00856. 28080049

[ref52] Xu X. PEPpy: A Tool to Generate Bacterial Peptidoglycan Scaffolds for Coarse-Grained Simulations, Doctoral dissertation, Syracuse University 2019.

[ref53] Bussi G.; Donadio D.; Parrinello M. Canonical Sampling through Velocity Rescaling. J. Chem. Phys. 2007, 126 (1), 14101 10.1063/1.2408420. 17212484

[ref54] Berendsen H. J. C.; Postma J. P. M.; van Gunsteren W. F.; DiNola A.; Haak J. R. Molecular Dynamics with Coupling to an External Bath. J. Chem. Phys. 1984, 81 (8), 3684–3690. 10.1063/1.448118.

[ref55] Parrinello M.; Rahman A. Polymorphic Transitions in Single Crystals: A New Molecular Dynamics Method. J. Appl. Phys. 1981, 52 (12), 7182–7190. 10.1063/1.328693.

[ref56] Du C.; Liu J.; Fikhman D. A.; Dong K. S.; Monroe M. B. B. Shape Memory Polymer Foams With Phenolic Acid-Based Antioxidant and Antimicrobial Properties for Traumatic Wound Healing. Front. Bioeng. Biotechnol. 2022, 10, 168 10.3389/fbioe.2022.809361. PMC8893234 35252129

[ref57] Feng P.; Jia J.; Yu L.; Min A.; Yang S.; Shuai C. Accelerated Degradation of Poly(l-Lactide) Bone Scaffold: Crystallinity and Hydrophilicity. Mater. Chem. Phys. 2021, 266, 124545 10.1016/j.matchemphys.2021.124545.

[ref58] Ru J.; Wang Z.; Tong C.; Liu H.; Wang G.; Peng Z. Nonleachable Antibacterial Nanocellulose with Excellent Cytocompatible and UV-Shielding Properties Achieved by Counterion Exchange with Nature-Based Phenolic Acids. ACS Sustain. Chem. Eng. 2021, 9 (47), 15755–15767. 10.1021/acssuschemeng.1c03561.

[ref59] Harish Nayaka M. A.; Sathisha U. V.; Dharmesh S. M. Cytoprotective and Antioxidant Activity of Free, Conjugated and Insoluble-Bound Phenolic Acids from Swallow Root (Decalepis Hamiltonii). Food Chem. 2010, 119 (4), 1307–1312. 10.1016/j.foodchem.2009.08.044.

[ref60] Gu H.; Lee S. W.; Buffington S. L.; Henderson J. H.; Ren D. On-Demand Removal of Bacterial Biofilms via Shape Memory Activation. ACS Appl. Mater. Interfaces 2016, 8 (33), 21140–21144. 10.1021/acsami.6b06900. 27517738 PMC5222513

