# Supplementary material for: Self-Defensive Antimicrobial Shape Memory Polyurethanes with Honey-Based Compounds
Source: ACS Appl Mater Interfaces. 2023 Dec 4;15(49):56733–48. doi: 10.1021/acsami.3c12274 (PMC10726308; doi:10.1021/acsami.3c12274)
Supplement: Supplementary file 1 — am3c12274_si_001.pdf [file am3c12274_si_001.pdf]

Supporting Information

Self-defensive antimicrobial shape memory polyurethanes with honey-based compounds

Maryam Ramezani,[1] Emily Elizabeth Labour,[1] Jingjing Ji,[1] Anand Utpal Vakil,[1] Changling Du,[1] Thalma Kabeyi Orado,[1] Shikha Nangia,[1] and Mary Beth Browning Monroe[1]*

[1]Department of Biomedical and Chemical Engineering, Syracuse Biomaterials Institute, and BioInspired Syracuse: Institute for Material and Living Systems, Syracuse University, Syracuse, NY 13244.

*Corresponding author: Dr. Mary Beth Browning Monroe

Department of Biomedical and Chemical Engineering, BioInspired Syracuse: Institute for Materials and Living Systems

Syracuse University

318 Bowne Hall

Syracuse, NY 13244

Tel: (315) 443-3323

E-mail: mbmonroe@syr.edu

1. **Chemical characterization of glycerol modification with phenolic acids. Figure S1** shows ^{1}H-nuclear magnetic resonance spectra of modified cinnamic (left) and p-coumaric (right) acids. The introduction of a shift at ~4.4-4.5 (B) after glycerol esterification was used to confirm successful synthesis with ~92% modification of glycerol with phenolic acids (standardized to ring shifts at ~7.4-7.5).

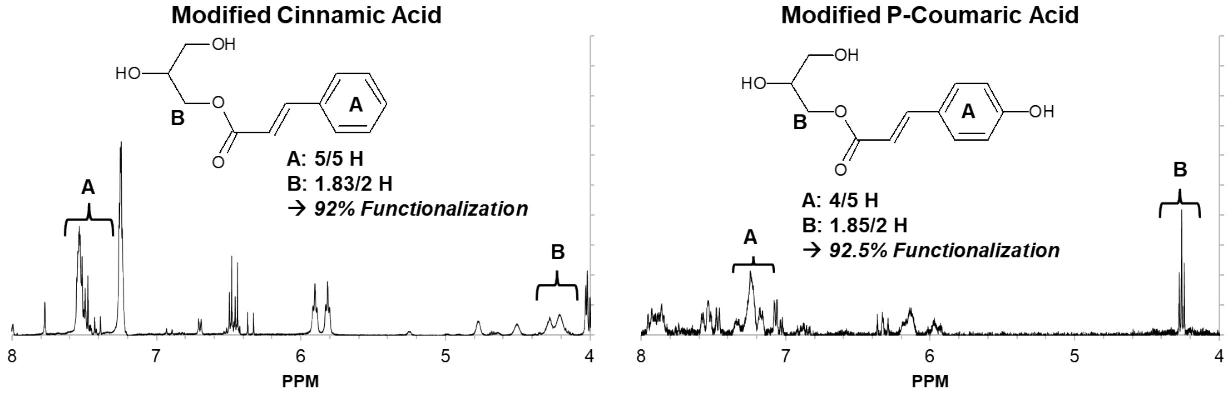

Figure S1. Nuclear magnetic resonance spectra of glycerol modified with cinnamic (left) and p-coumaric (right) acids.

2. Atomistic to CG mapping scheme. Figure S2 shows the MARTINI coarse grain models for CA: Cinnamic acid, PCA: p-coumaric acid, and FA: ferulic acid. **Table S1** specifies that MARTINI force field parameters for CA, PCA, and FA.

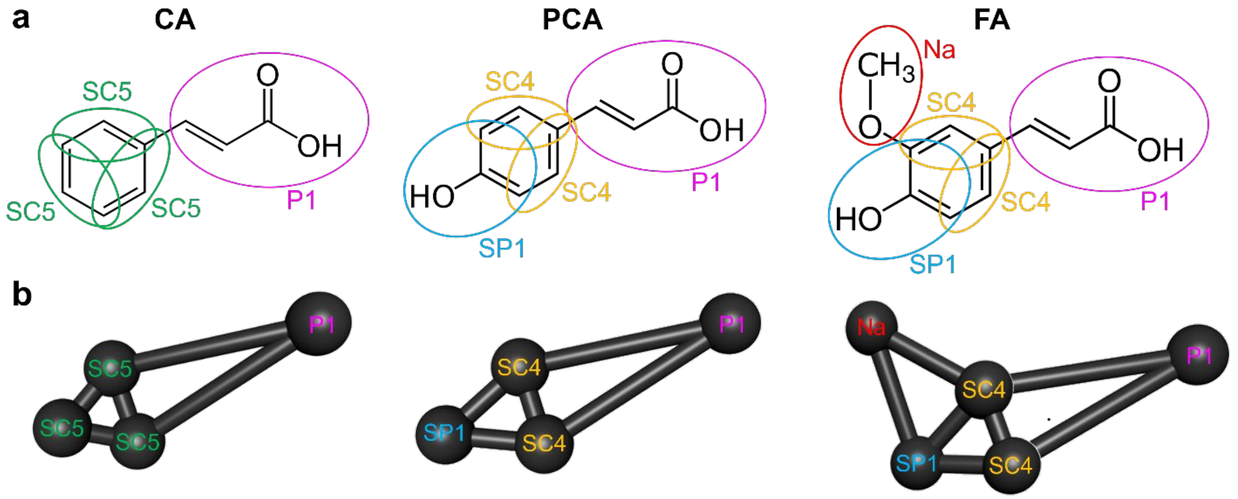

Figure S2. (a) The atomistic structures and (b) CG models of CA, PCA, and FA. The CG bead types P1, SC5, SC4, SP1, and Na are labeled in magenta, green, orange, cyan, and red, respectively.

Table S1. MARTINI force field parameters of CA, PCA, and FA.

[moleculetype]						
**CA**	1					
[atoms]						
1	SC5	1	39BI	C1	1	0
2	SC5	1	39BI	C2	2	0
3	SC5	1	39BI	C3	3	0
4	P1	1	39BI	Q1	4	0
[bonds]						
1	4	1	0.43409	15148.31703		
3	4	1	0.43343	16783.34576		
[angles]						
2	1	4	2	138.11657	301.84898	
2	3	4	2	138.73185	277.33155	
[constraints]						
1	2	1	0.16100			
2	3	1	0.16088			
1	3	1	0.16012			

[moleculetype]						
**PCA**	1					
[atoms]						
1	SC4	1	IB1E	C1	1	0

2	SP1	1	IB1E	C2	2	0
3	SC4	1	IB1E	C3	3	0
4	P1	1	IB1E	Q1	4	0
[bonds]						
1	4	1	0.43318	14939.16033		
3	4	1	0.43398	14112.59675		
[angles]						
2	1	4	2	144.18359	335.91541	
2	3	4	2	143.86617	372.95569	
[constraints]						
1	2	1	0.20112			
2	3	1	0.20058			
1	3	1	0.16146			

[moleculetype]						
**FA**	1					
[atoms]						
1	SC4	1	G432	C1	1	0
2	SP1	1	G432	C2	2	0
3	SC4	1	G432	C3	3	0
4	Na	1	G432	N1	4	0
5	P1	1	G432	Q1	5	0
[bonds]						
1	5	1	0.41127	1781.74449		
3	5	1	0.40730	1527.00021		
[angles]						
1	2	4	2	108.73192	2370.73487	
2	1	5	2	140.70911	114.52899	
2	3	5	2	142.06157	110.47144	
1	3	4	2	142.13692	4968.55539	
[constraints]						
1	2	1	0.20173			
2	3	1	0.20429			
1	3	1	0.16263			
4	2	1	0.30417			
4	3	1	0.27493			

3. Surface chemistry characterization. Figure S3 shows the FTIR results of polyurethanes with incorporated phenolic acids: CA: Cinnamic acid, PCA: p-coumaric acid, MPCA: modified PCA, FA: ferulic acid.

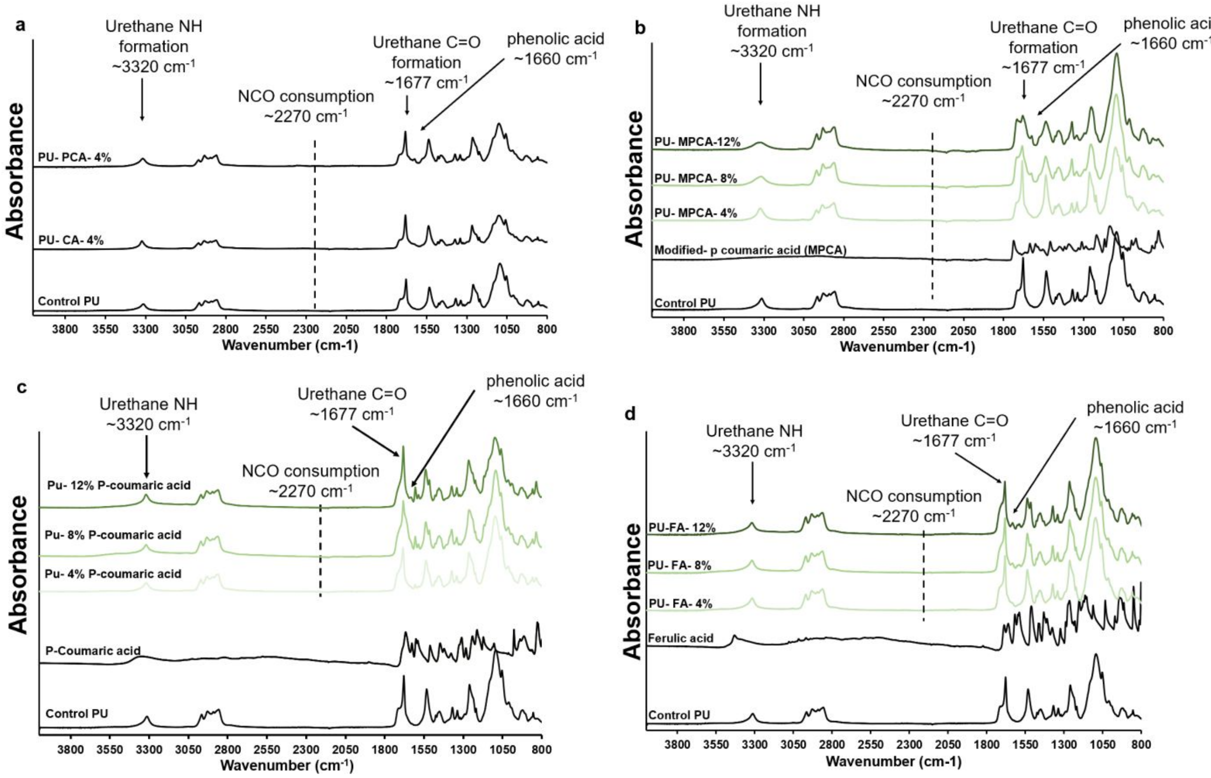

Figure S3. FTIR spectra of polyurethanes with a) chemical incorporation of unmodified CA and PCA, b) chemical incorporation of modified PCA, c) physical incorporation of PCA, and d) physical incorporation of FA.

4. Thermal analysis of polyurethanes. Figure S4 shows the differential scanning calorimetry thermograms used to determine glass transition temperatures (Tg) and melting temperatures (Tm) of the hard segments of synthesized polymers.

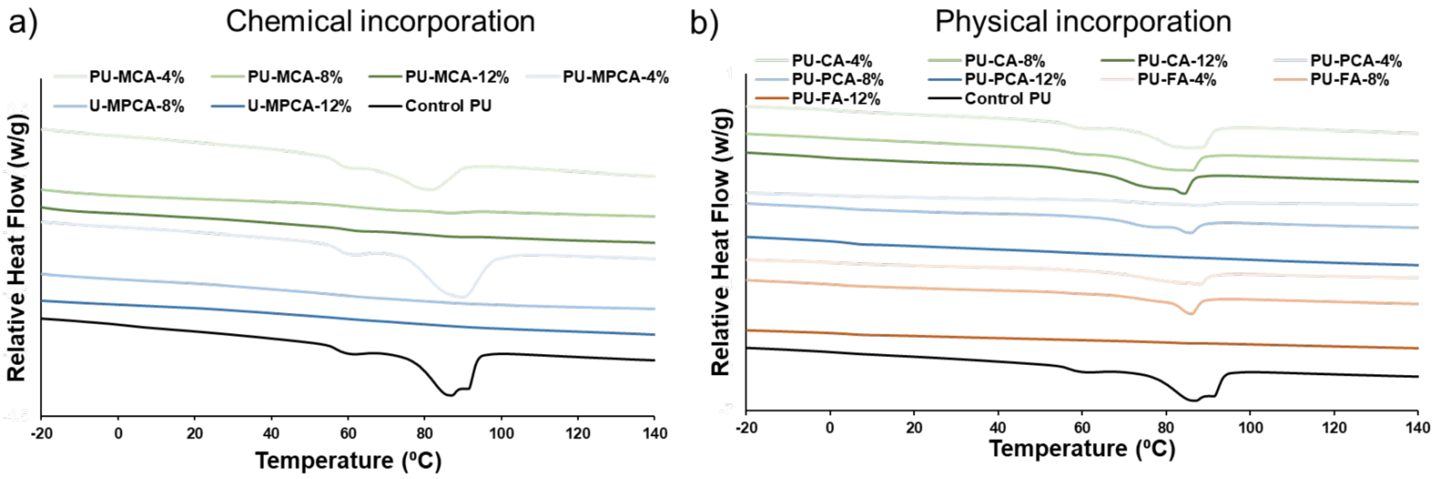

Figure S4. Differential scanning calorimetry thermograms of synthesized polymers with (a) chemically incorporated and (b) physically incorporated phenolic acids (endotherm down).

5. Mechanical analysis of polyurethanes. Figure S5 shows stress vs. strain curves obtained from tensile testing of synthesized polymers, which were used to calculate tensile strength, elongation at break, and modulus.

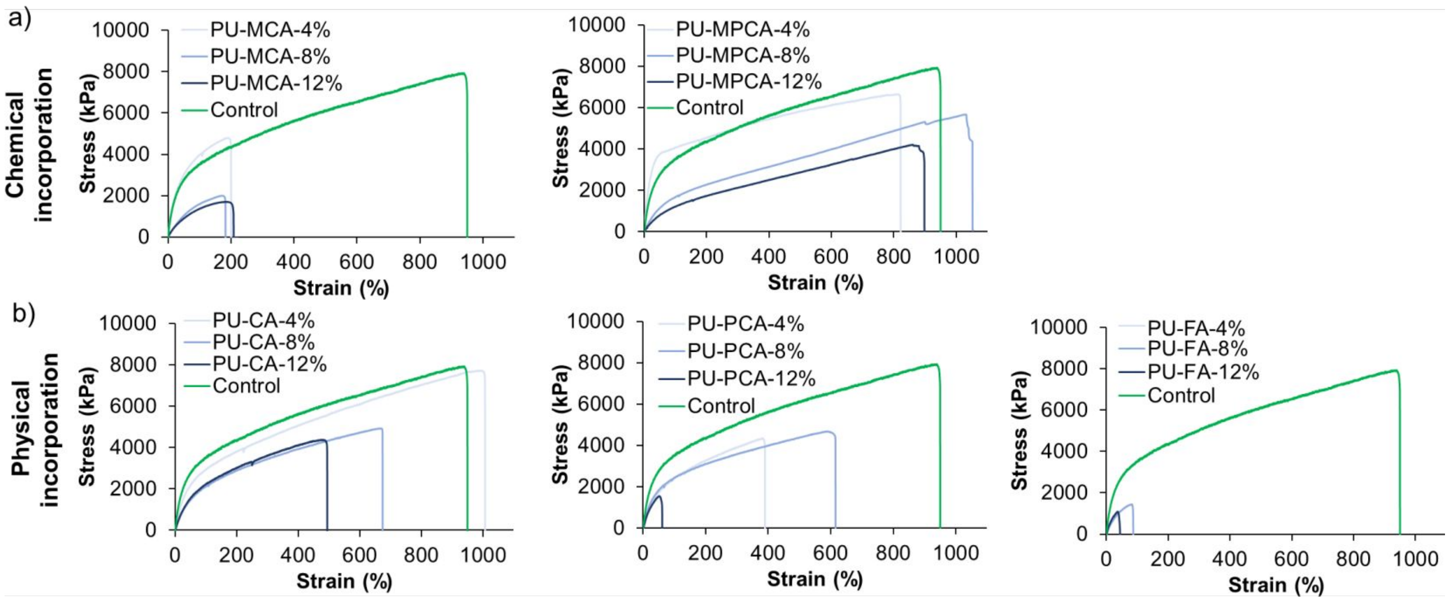

Figure S5. Stress vs. strain curves of synthesized polymers with (a) chemically incorporated and (b) physically incorporated phenolic acids.

6. Shape memory properties of polyurethanes. Figure S6 shows dynamic mechanical analysis plots obtained during shape memory testing of synthesized polymers, which were used to calculate shape fixity and shape recovery.

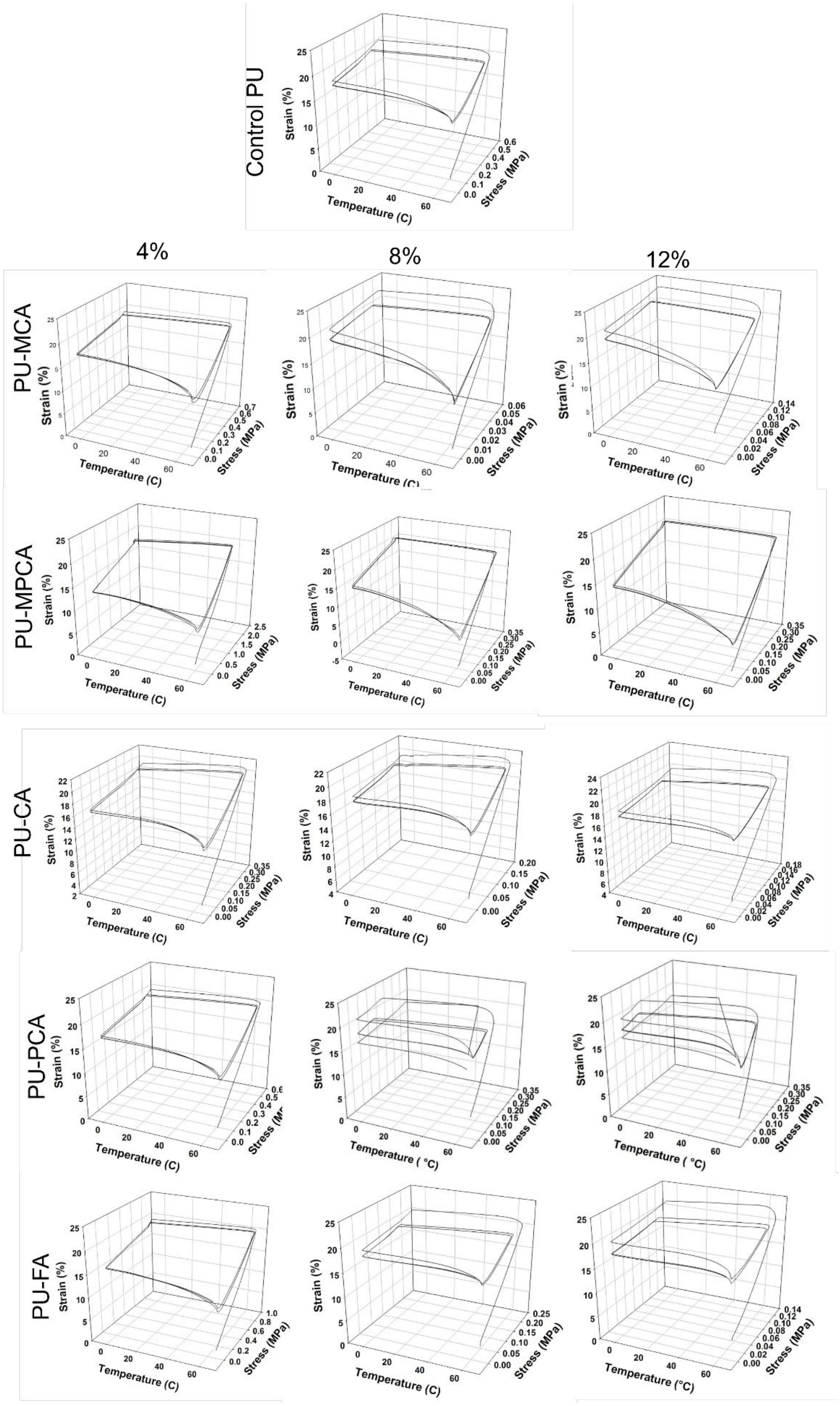

Figure S6. Dynamic mechanical analysis of shape memory properties of synthesized polymers over 3 cycles.

7. Thermomechanical, shape memory, and degradation properties of polyurethanes with direct chemical incorporation of unmodified CA and PCA. Table S2 shows that chemical incorporation of unmodified CA and PCA into the polyurethane resulted in weak and brittle polymers. In the case of CA, failure occurred during sample cutting with a dog bone punch, preventing mechanical characterization. This brittleness is attributed to termination of the polymer chains by CA and PCA, which only have one functional carboxylic acid group, that likely results in short chain polymers or oligomers during synthesis. In the case of PU-PCA-4%, we were able to obtain dog bone samples and run tensile testing. This effect may be due to the OH pendant group on PCA, which could aid in physical crosslinking. However, low elongation and tensile strength still show the relative brittleness of these polymers. PU-CA-4% and PU-PCA-4% (unmodified CA and PCA) do not exhibit shape memory properties due to these effects.

Table S2. Thermo-mechanical properties of synthesized PUs with **chemically** incorporated PAs. Mean ± standard deviation displayed. N = 1 for R_f and R_r; N = 3 for mechanical, and thermal properties.

Sample ID	R_f & R_r (%)	Mechanical Properties			Thermal Properties		
		Tensile Strength (kPa)	Elongation at Break (%)	Modulus (kPa)	T_g (°C)	T_m (°C)	ΔH (J/g)
Control	84 & 95	7600 ± 400	950 ± 3	98 ± 10	57 ± 1	87 ± 1	12 ± 1
PU-CA-4%	N/A	N/A	N/A	N/A	54 ± 0.5	78 ± 0.5	10 ± 6
PU-PCA-4%	N/A	1900 ± 200	6 ±1	150 ± 40	56 ± 0.4	84 ± 0.7	9 ± 0.4

As shown in **Figure S7 (upper)**, chemical incorporation of unmodified CA increases mass loss compared to polyurethanes with modified CA, which is attributed to brittleness of the polymer structure. On the other hand, chemical incorporation of unmodified and modified PCA resulted in reduced mass loss due to stronger interactions between PCA and polymer chains. **Figure S7 (lower)** shows FTIR and corresponding hard segment Tg of samples with chemically incorporated unmodified CA and PCA throughout 10 days of storage in PBS at 37°C. Surface chemistry and thermal properties remained stable in these polymers during this time frame.

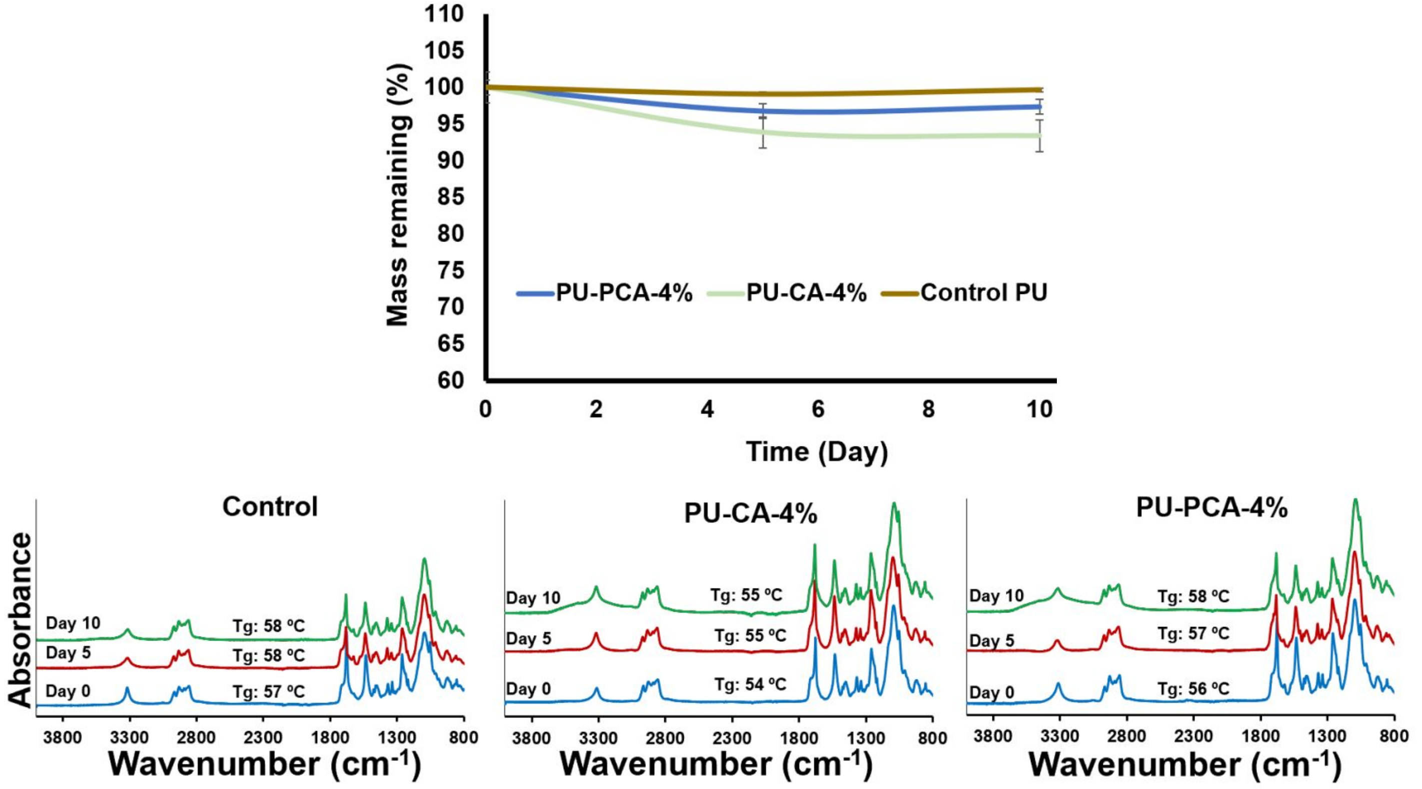

Figure S7. (Upper) Mass loss of polyurethanes (PU) with chemically incorporated unmodified PAs compared to control. **(Lower)** FTIR and corresponding T_g (N=1) of control polyurethane and samples with chemically incorporated unmodified PAs throughout 10 days of storage in PBS at 37°C.

8. Quantification of biomass: Crystal violet assay results for physical and chemical incorporation of PA into polyurethanes are shown in **Figure S8**. According to absorbance measurements, chemical and physical incorporation of PAs reduced surrounding biomass attached to the plate, with comparable results to those of the Ag foam clinical control. The Control PU had a relatively low biomass in surrounding areas, which was unexpected. One hypothesis for this result is that bacteria preferentially attach to the surface of the control PU vs. to the plate, resulting in lower biomass measurements in this assay.

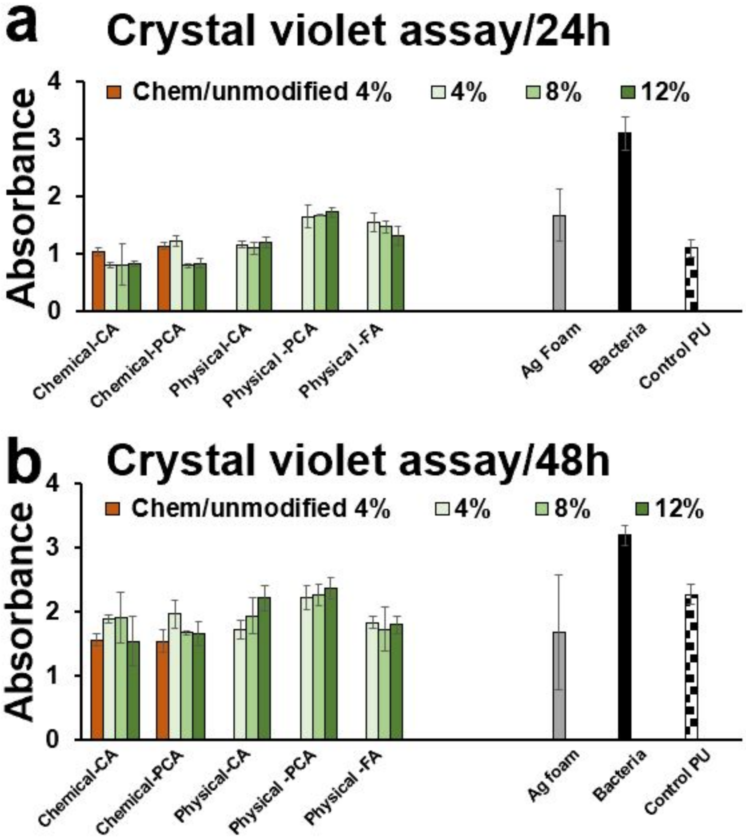

Figure S8. Crystal violet assay of wells surrounding polyurethane samples with physically and chemically incorporated phenolic acids after a) 24 and b) 48 hours.
